# Antenatal corticosteroids for pregnant women at risk of preterm labour in low- and middle-income countries: utilisation and facility readiness

**DOI:** 10.7189/jogh.15.04149

**Published:** 2025-05-23

**Authors:** Wen-Chien Yang, Catherine Arsenault, Victoria Y Fan, Hannah H Leslie, Fouzia Farooq, Andrea B Pembe, Theodros Getachew, Emily R Smith

**Affiliations:** 1 Department of Global Health, Milken Institute School of Public Health, The George Washington University, Washington, D.C., USA; 2 Center for Global Development, Washington, D.C., USA; 3 College of Social Sciences, University of Hawai‘i at Mānoa, Honolulu, Hawaii, USA; 4 Division of Prevention Science, University of California San Francisco, San Francisco, California, USA; 5 School of Clinical Medicine, Muhimbili University of Health and Allied Sciences, Dar es Salaam, Tanzania; 6 Ethiopian Public Health Institute, Addis Ababa, Ethiopia; 7 Department of Exercise and Nutrition Sciences, Milken Institute School of Public Health, The George Washington University, Washington, D.C., USA

## Abstract

**Background:**

Administering antenatal corticosteroids (ACS) to pregnant women at risk of imminent preterm labour improves newborn survival. However, ACS remains substantially underused in low- and middle-income countries (LMICs), where most preterm births occur globally. Providing ACS in inadequately equipped settings can be harmful. Health facilities must demonstrate readiness to ensure safe and effective ACS use. We aimed to assess ACS utilisation and facility readiness to administer ACS based on the World Health Organization (WHO) recommendations.

**Methods:**

We used data from Service Provision Assessments in nine LMICs. The primary outcome was ACS utilisation, which was defined as having ever provided ACS in a health facility. We assessed the availability of injectable corticosteroids (dexamethasone or betamethasone) and facility readiness to administer ACS appropriately. To measure readiness, we developed an overall readiness index based on 35 indicators, grouped into four categories based on WHO recommendations. The results were stratified by facility level.

**Results:**

Across eight countries with comparable sampling strategies, only a median of 10.7% (range = 6.7–35.2%) of facilities had provided ACS, one-fourth (median = 25.3%; range = 4.6–61.5%) had injectable corticosteroids available at the time of the survey. Significant gaps were observed between corticosteroid availability and ACS use. We found low overall readiness indices, ranging from 8.1% for Bangladesh to 32.9% for Senegal. Across four readiness categories, the readiness index was the lowest for criterion one (*i.e.* ability to assess gestational age accurately) (7.3%), followed by criterion two (*i.e.* ability to identify maternal infections) (24.8%), criterion four (*i.e.* ability to provide adequate preterm newborn care) (31.3%), and criterion three (*i.e.* ability to provide adequate childbirth care) (32.9%).

**Conclusions:**

We proposed a strategy for measuring facility readiness to implement one of the most effective interventions to improve neonatal survival. Countries should operationalise readiness measurement, improve facility readiness to provide ACS appropriately, and encourage ACS uptake in well-equipped facilities.

Antenatal corticosteroids (ACS) use among pregnant women at risk of imminent preterm labour is one of the most effective interventions for improving neonatal survival. By accelerating fetal lung maturity, ACS can reduce the risks of respiratory distress syndrome by 30% and neonatal death by 20% [1]. The World Health Organization (WHO) and other professional medical organisations recommend administering ACS to pregnant women with a high likelihood of preterm labour at gestational age (GA) of 24–34 weeks [2–4].

However, ACS adoption remains extremely limited in low- and middle-income countries (LMICs) despite these countries accounting for 80% of preterm births globally [5]. An estimated 13.4 million newborns were born prematurely in 2020, with preterm birth being the leading cause of neonatal death and accounting for nearly half (46%) of under-five mortality [6,7]. In addition, 75% of neonatal deaths occur within the first week of life, with one million neonatal deaths happening within the first 24 hours [8,9]. These statistics highlight the urgency of prioritising interventions that target the vast population of preterm babies [10,11]. ACS has been considered ‘the lowest-hanging and sweetest fruit’ as an intervention for improving preterm outcomes in LMICs [12]. While ACS use has been widely advocated in LMICs, its uptake in these countries remains a subject of ongoing discussions, and evidence on the current landscape of ACS use in resource-constrained countries is still scarce [13–17].

Two significant evidence gaps remain. First, the current status of ACS use in LMICs is mainly unknown despite outdated coverage data. Second, the structural readiness of health facilities in LMICs to provide ACS following international guidelines is unclear. We defined facility readiness based on the WHO statement that facilities should ensure the availability of components required to provide health services, such as equipment, medicines, diagnostics, and commodities [18]. In 2022, the WHO released its updated recommendations, emphasising five conditions for safe and effective administration of ACS: GA can be accurately assessed, there is a high likelihood of preterm birth within seven days of starting ACS therapy, there is no evidence of maternal infections, adequate childbirth care is available, and the preterm newborn can receive appropriate care [2].

The WHO recommendations caution that these five conditions may not be met consistently across settings due to variations in facility capabilities, highlighting the potential harms of ACS in health care settings that cannot meet the criteria [2]. As a potent anti-inflammatory drug, ACS suppresses immune functions. Maternal infections remain a significant concern if ACS is given to vulnerable pregnant women who are ineligible for this intervention. Also, observational studies have reported increased neurocognitive disorders among late preterm infants (*i.e.* born at GA 34–36 weeks) who were exposed to ACS [19–21]. The balance between the benefits and risks emphasised the importance of locations where ACS should be provided. However, knowledge about facility readiness in resource-constrained countries remains limited. In 2021, Kankaria et al. found that primary and secondary facilities in northern India were not ready to administer ACS safely, which is, to date, the only study assessing facility readiness to give ACS [22]. As facility readiness is crucial for delivering health care of good quality [23], there is an urgent need to comprehensively understand the readiness of health facilities in LMICs to implement this life-saving intervention appropriately.

To address these critical evidence gaps, we aimed to assess ACS utilisation, corticosteroid availability, and facility readiness to administer ACS according to the WHO recommendations.

## METHODS

### Study sample

We used Service Provision Assessment (SPA) data, a health facility survey on service availability and quality of care in LMICs [24]. We restricted our analyses to SPA surveys conducted in the past ten years and used the latest survey available for countries with SPA data. We included ten surveys from nine countries: Afghanistan 2018–19, Bangladesh 2017–18, Nepal 2021, Haiti 2017–18, Democratic Republic of the Congo (DRC) 2017–18, Ethiopia 2021–22, Malawi 2013–14, Senegal 2018 and 2019, and Tanzania 2014–15. All surveys were completed before the release of the 2022 WHO recommendations, and most surveys, except Malawi 2013–14 and Tanzania 2014–15 surveys, were conducted after the WHO recommendations on interventions to improve preterm birth outcomes in 2015, which included guidelines on ACS use [25]. The sampling strategies varied based on country needs (Table S1 in the **Online Supplementary Document**) [26–35]. Most surveys adopted stratified random sampling strategies to obtain a nationally representative sample in the country [26–28,31,32]. Malawi 2013–14 and Haiti 2017–18 SPA surveys were national censuses [33,35]. Unlike other surveys, Afghanistan 2018–19 mainly sampled urban hospitals (public and private) and private clinics [34]. Senegal implemented continuous SPA over five consecutive years to survey all health facilities [29,30,36]. To have similar sample sizes across countries, we merged data for Senegal for two years (2018 and 2019). We used data from two core instruments (out of five) of the SPA survey questionnaire: facility inventory and health worker interviews. In each country, we only included facilities that provided antenatal care (ANC) or delivery services (*i.e.* regular deliveries or caesarean deliveries).

### Measures

Our primary outcome was ACS utilisation, defined as facilities having ever provided ACS to pregnant women. We focussed on two secondary outcomes: corticosteroid availability (*i.e.* injectable dexamethasone or betamethasone) and facility structural readiness. In SPA surveys, most countries did not assess corticosteroid availability within the maternal and newborn care section, except for the Afghanistan 2018–19 survey. Alternatively, we used the availability of injectable corticosteroids in the section for medicines for non-communicable diseases as a proxy.

Following the 2022 WHO recommendations on ACS use, we assessed facility readiness to provide ACS. From the SPA questionnaire, we identified 35 essential items (including equipment, medicines, diagnostics, commodities, and staff) required to fulfil the criteria for appropriate ACS provision. We grouped these 35 items, used as indicators to assess readiness, into four readiness categories based on four of the five WHO criteria (Table S2 in the **Online Supplementary Document**). We did not identify indicators for the second criterion – a high likelihood of preterm labour, because the diagnosis of imminent preterm labour is primarily based on clinical symptoms and signs, including preterm membrane rupture without labour or spontaneous preterm labour (*i.e.* at least six regular uterine contractions per hour plus cervical dilatation ≥3 cm or effacement ≥75%) [2]. The first readiness category focussed on the facility’s ability to assess GA accurately, and we included only one indicator for this category (*i.e.* the presence of a functional ultrasound) according to the WHO recommendations about accurate GA assessment [2]. The second readiness category included four indicators and covered the facility’s ability to identify maternal infections. The third and fourth categories included 13 and 17 indicators and assessed the facility’s readiness to provide adequate childbirth and preterm newborn care, respectively. Based on the WHO recommendations on the appropriate settings for ACS administration, we identified the indicators for adequately performing comprehensive emergency obstetric care to assess the readiness of providing adequate delivery care, and the indicators about newborn resuscitation, management of respiratory distress, thermal care, and blood glucose monitoring to evaluate the ability for providing adequate preterm newborn care [2]. For each category, we calculated readiness indices by dividing the number of indicators available by the total number of indicators assessed, with higher percentages indicating higher readiness. We calculated an overall readiness index by averaging the readiness indices from four categories. We chose this approach based on previous studies on facility readiness to implement health improvement interventions [37,38].

### Statistical analysis

First, we presented descriptive statistics of ACS utilisation, corticosteroid availability, and facility readiness indices, taking facility sampling weights into account. We stratified the results by three facility levels. Level 1 facilities only provided ANC and did not conduct deliveries; ANC was defined as the care that pregnant women receive before birth, including risk identification, prevention, and management of pregnancy-related health conditions, education, and health promotion [39]. Level 2 facilities performed regular but not caesarean deliveries, while level 3 facilities performed caesarean deliveries. Data for Afghanistan were reported separately, because the country mainly sampled urban hospitals (both public and private) and private clinics, which differed substantially from other countries. We excluded Afghanistan from the distribution of outcomes across countries and included it only in analyses within each facility level.

Second, we assessed the relationships between ACS use and corticosteroid availability and between ACS use and overall readiness indices at sub-national levels (*e.g.* regions in Ethiopia and provinces in DRC). This approach was driven by the hypothesis that facilities without corticosteroids or low readiness might refer patients needing ACS to nearby facilities. We averaged the three measures (*i.e.* ACS use, corticosteroid availability, and overall readiness index) for all facilities in each sub-national region. Lastly, we examined the differences in the overall readiness index between facilities that had ever and never used ACS by country. All analyses were performed using *R*, version 4.4.0. (R Core Team, Vienna, Austria).

## RESULTS

We included 8669 facilities from ten surveys in nine countries in the analysis. All surveys had a high response rate (median = 94.9%), ranging from 88.8% in Afghanistan to 99.0% in Tanzania (Table S3 in the **Online Supplementary Document**).

Most facilities (median = 88.9%; range = 66.1–98.8%) provided maternal health care services and were included in our analyses. The median sample size was 929 facilities (range = 108–1500) (**Table 1**). Among eight countries (excluding Afghanistan), 22.6% (range = 7.1–88.7%) of facilities were urban. The proportion of facilities at different levels varied across countries. Across eight countries, one-third (median = 32.0%) of the facilities were level 1 facilities that provided ANC, with Bangladesh having the largest proportion (76.2%) of level 1 facilities and the DRC having the smallest proportion (1.9%). The proportion of level 2 facilities was 59.4%, with Tanzania having the largest proportion (83.3%) and Bangladesh having the smallest proportion (19.5%). Only 6.2% of facilities were level 3 facilities, ranging from 2.7% in Ethiopia to 26.5% in DRC facilities.

**Table 1:** Characteristics of surveys and facilities included in the study

					Facility type, n (%)		
	**Survey year**	Number of facilities surveyed (n = 9793)*	**Number of facilities included (n = 8669)†**	**Urban location, n (%)**	**Level 1: provide ANC**	**Level 2: perform regular delivery**	**Level 3: perform caesarean section**
**Median (range)**		1158 (142–1576)	929 (108–1500)	22.6 (7.1–88.7)‡	32.0 (1.9–76.2)‡	59.4 (19.5–83.3)‡	6.2 (2.7–26.5)‡
**South Asia**							
Afghanistan	2018–19	142	108	107 (99.4)	7 (4.1)	10 (10.1)	91 (85.9)
Bangladesh	2017–18	1524	1498	379 (7.1)	676 (76.2)	551 (19.5)	271 (4.4)
Nepal	2021	1576	1500	961 (53.2)	693 (47.5)	565 (47.1)	242 (5.3)
**Caribbean**							
Haiti	2017–18	1007	929	340 (36.5)	567 (61.0)	255 (27.5)	107 (11.5)
**Sub-Saharan Africa**							
DRC	2017–18	1380	1364	297 (22.0)	10 (1.9)	511 (71.6)	843 (26.5)
Ethiopia	2021–22	1158	911	455 (18.2)	261 (75.0)	309 (22.3)	341 (2.7)
Malawi	2013–14	977	645	119 (18.6)	103 (16.5)	471 (72.6)	71 (10.9)
Senegal	2018, 2019	841	658	594 (88.7)	65 (14.3)	531 (78.6)	62 (7.1)
Tanzania	2014–15	1188	1056	325 (19.1)	104 (11.5)	681 (83.3)	271 (5.2)

ANC – antenatal care, DRC – Democratic Republic of the Congo

*A total of 625 sampled facilities had a sampling weight of zero, indicating non-response, refusal, or closure. Those facilities were not surveyed and were excluded.

†Only facilities that provided either ANC or delivery services (regular delivery or caesarean section) were included in the analysis.

‡The medians and ranges were obtained across eight countries, excluding Afghanistan, because its survey used a remarkably different sampling strategy.

### ACS utilisation

**Figure 1:**
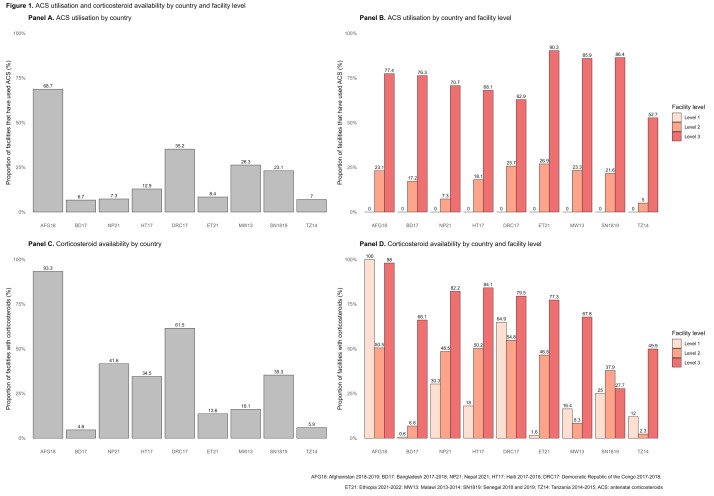
ACS utilisation and corticosteroid availability by country and facility level. **Panel A.** ACS utilisation by country. **Panel B.** ACS utilisation by country and facility level. **Panel C.** Corticosteroid availability by country. **Panel D.** Corticosteroid availability by country and facility level. ACS – antenatal corticosteroids.

ACS was underused across all countries. Excluding Afghanistan, the median ACS use was 10.7%, ranging from 6.7% of facilities in Bangladesh to 35.2% of facilities in the DRC that had ever administered ACS (**Figure 1**, Panel A). In Afghanistan, 68.7% of facilities had administered ACS during the survey. We did not find higher ACS use in surveys that were done more recently. While none of the level 1 facilities had provided ACS, ACS use increased by facility level (**Figure** 1, Panel B). Within each level of facilities across nine countries, 21.6% (range = 5.0–26.9%) of level 2 facilities and 76.3% (range = 52.7–90.3%) of level 3 facilities had administered ACS.

### ACS availability

Corticosteroid availability was limited. Across eight countries, one-fourth of the facilities (median = 25.3%; range = 4.6–61.5%) had at least one valid corticosteroid (injectable dexamethasone or betamethasone) available at the time of the survey, while 93.3% of facilities in Afghanistan had it available (**Figure 1**, Panel C). Corticosteroid availability generally increased by facility level within each country (**Figure 1**, Panel D). Notably, significant gaps existed between corticosteroid availability and ACS utilisation, particularly in level 2 facilities. Among level 2 facilities across nine countries, 46.5% had injectable corticosteroids available, but 21.6% had administered ACS (Figure S1 in the **Online Supplementary Document**).

### Facility readiness

**Figure 2:**
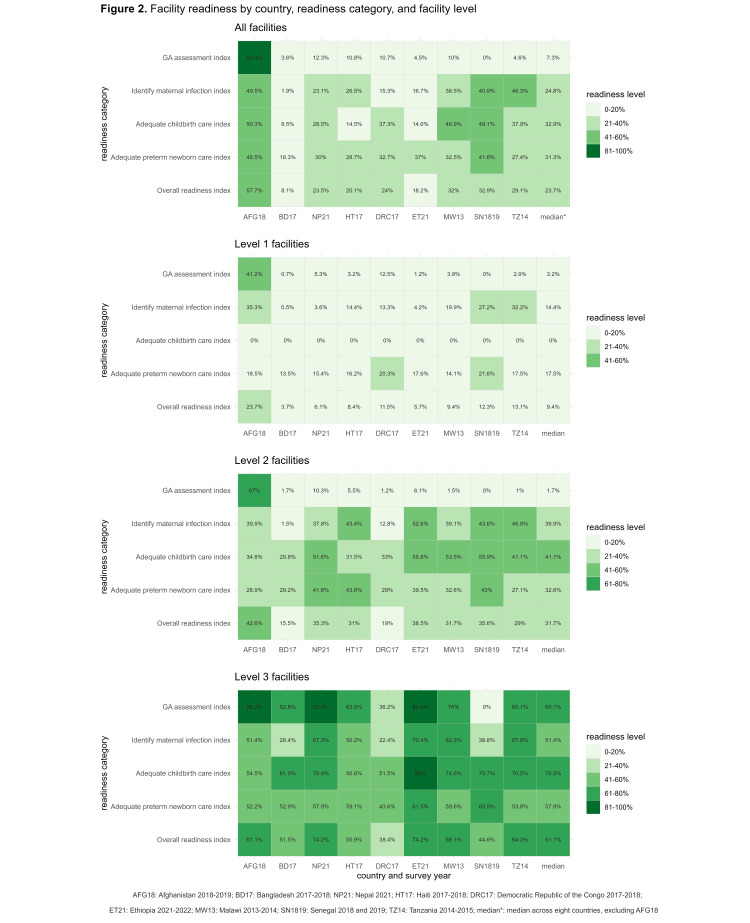
Facility readiness by country, readiness category, and facility level.

Readiness indices were low (**Figure 2**). Only 22% of the facilities in the sample had an overall readiness index above 50%. Other than Afghanistan, overall readiness indices were low among the eight countries, ranging from 8.1% in Bangladesh to 32.9% in Senegal. Afghanistan had an overall readiness index of 57.7%. We did not see higher overall readiness indices in surveys conducted in more recent years. Across four readiness categories among eight countries, facilities performed the worst in the ability to assess GA accurately, with a readiness index of 7.3%, followed by the ability to identify maternal infection (median = 24.8%), provide adequate preterm care (median = 31.3%), and provide adequate childbirth care (median = 32.9%). In addition, overall readiness indices increased at the facility level. The readiness indices for accurate GA assessment remained low for both level 1 and 2 facilities but increased for level 3 facilities. In contrast, indices for other categories consistently improved at the facility level. When delving into the details of specific indicators among the 35 indicators we identified, we found minimal availability for respiratory support-related equipment across countries in the readiness category of adequate preterm newborn care (Figures S2–5 and Table S4 in the **Online Supplementary Document**).

### ACS use, availability, and readiness at sub-national levels

**Figure 3:**
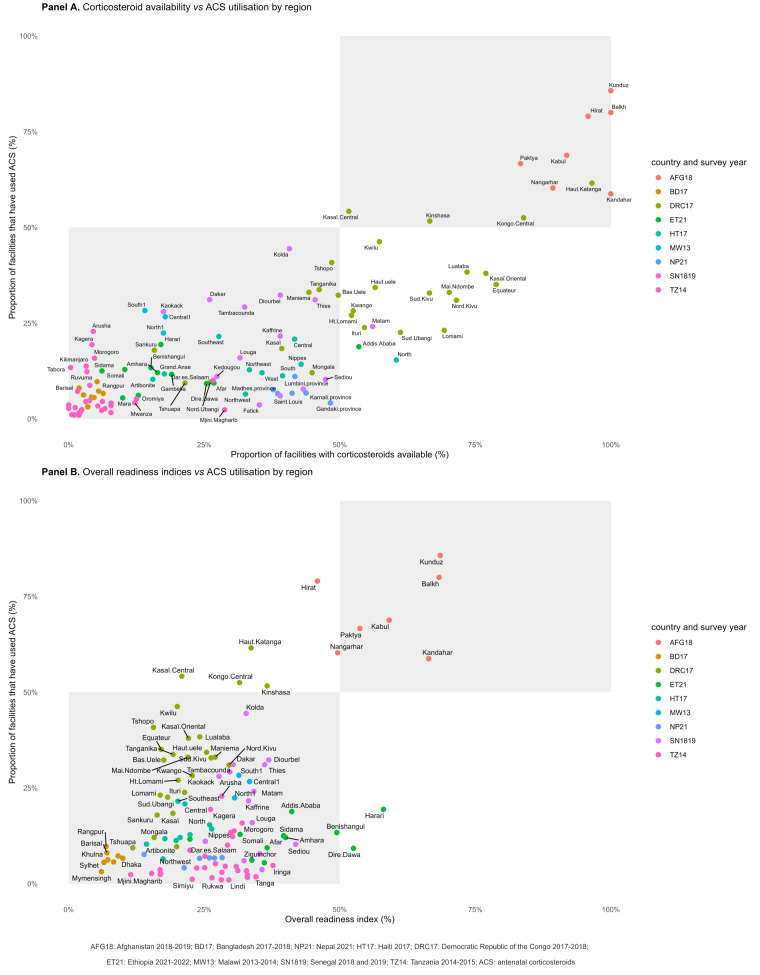
Relationships between corticosteroid availability, facility readiness, and ACS utilisation by region. **Panel A.** Corticosteroid availability *vs* ACS utilisation by region. **Panel B.** Overall readiness indices *vs* ACS utilisation by region. ACS – antenatal corticosterioids.

At the sub-national level, positive associations were observed between corticosteroid availability and ACS use and between overall readiness indices and ACS use (**Figure 3**, Panels A and B). A few Ethiopian regions (Dire Dawa and Harari) had an average overall readiness index above 50% but low ACS use (<25%). In contrast, some regions of the DRC (Kasaï Central, Kongo Central, Kinshasa, and Haut-Katanga) had low overall readiness indices (<50%), but ACS use was greater than 50% (**Figure 3**, Panel B).

At the facility level, overall readiness indices differed between facilities that had ever and never provided ACS within each country (Figures S6–8 in the **Online Supplementary Document**). In most countries (Afghanistan, Bangladesh, DRC, Malawi, Senegal, and Tanzania), the median overall readiness index for facilities (level 2 and level 3) that had utilised ACS was higher than that for facilities that had never used it. In contrast, the results were reversed for Nepal, Haiti, and Ethiopia (Figure S6 in the **Online Supplementary Document**).

## DISCUSSION

This study assessed ACS use, corticosteroid availability, and structural readiness to implement the intervention appropriately based on international recommendations among 8669 health facilities from nine LMICs. We had three major findings: ACS were substantially underused, limited corticosteroid availability, and facilities in these countries had low readiness levels.

We found limited ACS use, with only one out of ten health facilities having administered ACS. However, comparing our results with existing literature is challenging since previous research mostly measured ACS coverage among pregnant women who had preterm birth or antenatal exposure to ACS among preterm babies [40–45]. A WHO maternal and newborn health survey for 29 countries in 2011 found that 54% of women who gave birth at GA 26 to 34 weeks were given ACS, with the lowest use in Afghanistan (16%), Nepal (20%), and the DRC (16%) [41]. Another analysis using the data from the 2015 Antenatal Corticosteroids Trial (ACT trial) showed an overall low ACS use in control clusters; in Kenya, only 3.8% of pregnant women of infants born with birthweight less than the fifth percentile, a proxy for preterm births, received ACS [40]. Our findings are crucial to understanding the status of ACS utilisation at the facility level. In addition, we found that only one-fourth of the facilities in the sample had injectable dexamethasone or betamethasone. While dexamethasone is on the WHO List of Essential Medicines [46], its suboptimal availability remains a barrier to ACS uptake in countries that might benefit most from this intervention [47]. Some reasons for limited availability are contextual constraints, including an unstable supply chain, an insufficient inventory tracking and monitoring system, and varied national recommendations; health system strengthening is required to address these barriers [47–49]. Notably, we observed significant gaps between corticosteroid availability and ACS utilisation, highlighting great potential to prioritise readiness improvement initiatives among facilities where injectable corticosteroids are available. Interestingly, we did not observe more ACS utilisation or higher readiness in surveys conducted in recent years, but we lack repeated surveys in the same context, making it difficult to interpret the lack of a temporal trend.

Unlike the other eight countries, Afghanistan had higher ACS utilisation (69%), corticosteroid availability (93%), and overall readiness index (58%). Although a gap between corticosteroid availability and ACS utilisation still existed, both measures were notably higher than in other countries. This difference might be primarily attributable to the facility sampling strategy; the 2018 Afghanistan SPA survey mainly sampled urban hospitals (public and private) and private clinics, with 86% classified as level 3 facilities. Afghanistan does not have national recommendations on ACS use [50].

The locations for administering ACS are crucial. Two landmark studies on the effects of ACS in LMICs presented conflicting results, indicating the importance of health care settings to implement this intervention [51,52]. The 2015 ACT trial, a cluster-randomised trial of a multifaceted intervention to promote ACS use in six countries, unexpectedly found increased neonatal deaths and suspected maternal infections among intervention clusters [51]. On the contrary, the WHO Antenatal Corticosteroids for Improving Outcomes in Preterm Newborns Trial (ACTION-I trial) in 2020, an RCT in five countries, showed ACS reduced neonatal deaths without increasing maternal bacterial infections [52]. These contradictory findings could partially be explained by the different settings of these two trials: the ACT trial was done in all levels of care, including clinics and primary care centres, whereas the ACTION-I trial included secondary or tertiary hospitals. The drastically different findings emphasised the importance of health care settings – more precisely, the readiness level of facilities required for safe and effective use of ACS. Particularly, the increased risk of suspected maternal infections in the ACT trial highlights the importance of administering ACS to eligible pregnant women without infections, which requires accurate identification of infection based on clinical symptoms and diagnostic test results, including blood and urine tests. Also, although the WHO recommendations suggest providing ACS in cases without maternal infections, the types of infections (*i.e.* bacterial infections or all kinds of infections) were not explicitly described, which requires clear definitions for operational purposes.

Recommendations on what levels of facilities should administer ACS need to be made carefully. First, national guidelines and policies matter fundamentally. Some countries might not have guidelines on ACS use, while others with guidelines might differ in specifying the levels of care at which ACS should be given. The national guidelines affect the specific efforts to promote appropriate ACS utilisation across health system tiers and between countries. One policy analysis on ACS use in Africa found that it was recommended at lower levels of care before referral in Ethiopia and Tanzania, but the DRC and Malawi only allowed ACS use in hospitals [49]. Assuming essential equipment, medicines, and trained staff are available, some level 2 and level 3 facilities with high levels of readiness that have never provided ACS should be targeted for encouraging ACS use. Also, our sub-national level analyses found that some provinces in the DRC with low overall readiness (<50%) frequently administered ACS. In contrast, facilities in some Ethiopian regions had overall readiness indices above 50% but rarely administered ACS. Further studies should investigate the reasons for this type of discordance. Policymakers should ensure that ACS is delivered in well-equipped settings for safe and effective use and target facilities with higher readiness levels to promote ACS use.

Another critical issue centres around the different aspects of facility readiness. Our readiness index was developed based on the WHO criteria. However, it is debatable, first, whether some criteria could be met across facility levels, and second, whether facilities must meet all criteria to safely and effectively administer ACS. For example, ultrasound examination in early pregnancy is the gold standard for GA assessment. Nonetheless, our findings indicated that merely 7.3% (IQR = 0.2–12.3) of maternal care facilities across eight countries possessed a functional ultrasound. In contrast, 82.4% of facilities in Afghanistan were equipped with a functional ultrasound. In LMICs, limited access to obstetric ultrasound for GA dating remains a significant barrier to proper ACS use despite its increasing use [16,48]. In this case, GA dating should occur early in pregnancy, while ACS use occurs later in pregnancy. Thus, the decision to administer ACS should not be based on the ultrasound availability in place but on the availability of accurate GA assessment (which may come from care obtained at another facility). Another crucial element for adequate preterm newborn care from the WHO recommendations is non-invasive respiratory support. This recommendation might exclude most preterm newborns who will likely benefit from ACS exposure because they usually lack access to such respiratory support at the primary level of care in LMICs [53]. A similar concern applies to the criteria for adequate childbirth care, referring to nine Comprehensive Emergency Obstetric and Newborn Care signal functions, including blood transfusions and caesarean sections. Again, whether a facility can give ACS only when it can do blood transfusions is debatable. LMICs should consider improving readiness in lower-level facilities for safe and effective ACS use. Our findings also contribute to the ongoing discussion on the appropriate health system level where ACS should be provided. Ultimately, maintaining stable corticosteroid availability and improving facility readiness are crucial, but do not guarantee ACS use. Effective ACS utilisation requires efforts from multiple levels, including competent providers with the necessary knowledge and skills, an effective health service delivery structure with a strong referral system to ensure eligible women receive ACS and health services in appropriate settings, and fundamental support from national policy, guidelines, and implementation strategies. For example, recent discussions on service delivery redesign that brings pregnant women closer to higher levels of care might better ensure the benefits of ACS [54].

Our study has several strengths. This is the first study to comprehensively assess ACS utilisation, corticosteroid availability, and structural readiness to provide ACS at the facility level. We leveraged comparable, nationally representative data across eight countries, highlighting the strength of this study; our results helped to understand the landscape of ACS use and identified policy directions for areas with a high burden of preterm births. Our study also has a few limitations. First, sampling strategies varied across countries. Afghanistan primarily sampled hospitals (public and private) and private clinics, whereas the other eight countries obtained nationally representative samples of health facilities through stratified random sampling. Thus, Afghanistan data are not comparable with other countries, and we reported the pooled results excluding Afghanistan and refrained from cross-country comparisons. Second, the countries included in this study had different contexts. They varied in infrastructure, health systems, national priorities, and national guidelines on ACS use, all affecting ACS utilisation [49]. The survey years also spanned nine years. Differences by country likely reflect both distinct health systems and changes over time. Our analyses cannot fully disentangle the temporal changes from cross-country differences. Future studies might track trends and address this limitation. Therefore, we caution careful interpretations for any attempts to make cross-country comparisons, as we aimed to provide an overview of the landscape of ACS use at the facility in low-resource countries rather than drawing comparisons between countries. Third, some readiness indicators served as proxies because SPA surveys do not assess the availability of injectable corticosteroids and ultrasound in the maternal and newborn care section. The true availability of corticosteroids in the maternal care section might be lower than what we reported, as it is unlikely that drugs would be available in the ANC or labour and delivery sections but not in the central pharmacy or elsewhere. We also used the availability of rapid diagnostic tests for HIV and syphilis to estimate facilities’ ability to detect maternal infections. We recognise that this approach might overestimate or underestimate the actual availability or readiness, potentially affecting the validity of our findings. Future studies should avoid using proxies in assessing readiness, which could be achieved by adding new ACS-specific indicators to standard surveys such as SPA. Fourth, while we found positive relationships between ACS utilisation and corticosteroid availability and readiness, we acknowledge that these ecological associations may be influenced by several additional factors, including social, national, facility, provider, and patient characteristics that were not considered in the analysis. Previous studies have identified provider- and patient-level factors affecting ACS use, including perspective and provider perceptions of risks and benefits [48]. Although our focus was on the health system, future research should also consider sociocultural factors influencing ACS use to understand the whole picture of ACS utilisation in LMICs. The association between ACS availability and ACS use may also be biased since utilisation relates to the time before the survey. In contrast, availability is based on the presence of the drug at the time of the survey.

## CONCLUSIONS

ACS utilisation in LMICs has gained tremendous international attention as its health benefits at the population level could be profound [11,55–58]. The substantial underuse of ACS in LMICs is a huge missed opportunity; ethically, not providing an evidence-based intervention that has substantial potential to improve neonatal survival in areas where most preterm births happen fails to meet two key ethical criteria for health resource allocation: cost-effectiveness and equitable distribution of services [59]. ACS is promising but should never be used as a ‘just-in-case’ intervention [3]. To optimise the benefits of ACS and avoid potential harms, ACS must be given to the right people (pregnant women at risk of imminent preterm labour and without maternal infections), at the right time (the specified GA window), and in the right place (settings that are adequately equipped and ready to provide quality maternal and newborn care). Future research and programs should operationalise readiness measurement, enhance facility readiness to administer ACS, and encourage ACS uptake among well-equipped facilities.

## Additional Material


Online Supplementary Document
