# Supplementary material for: Antenatal corticosteroids for pregnant women at risk of preterm labour in low- and middle-income countries: utilisation and facility readiness
Source: J Glob Health. 2025 May 23;15:04149. doi: 10.7189/jogh.15.04149 (PMC12100574; doi:10.7189/jogh.15.04149)

Supplement to: Yang W-C, Arsenault C, Fan VY, Leslie HH, Farooq F, Pembe AB, Getachew T, Smith ER. Antenatal corticosteroids for pregnant women at risk of preterm labour in low- and middle-income countries: utilisation and facility readiness. J Glob Health. 2025;15:04149.

**Contents**		**Page**
**Table S1**	Sampling strategies of included SPA surveys	3
**Table S2**	The indicators used to assess facility readiness based on the 2022 WHO recommendations on ACS	4
**Table S3**	Response rates and number of facilities included in the analysis for each survey	5
**Table S4**	Facility structural readiness for 35 indicators by country and facility level	6
**Figure S1**	ACS utilization and corticosteroid availability by facility level	11
**Figure S2**	Heatmap of facility readiness for all facilities by country	12
**Figure S3**	Heatmap of facility readiness for level 1 facilities by country	13
**Figure S4**	Heatmap of facility readiness for level 2 facilities by country	14
**Figure S5**	Heatmap of facility readiness for level 3 facilities by country	15
**Figure S6**	Differences in overall readiness indexes by antenatal corticosteroids utilization for level 2 and level 3 facilities	16
**Figure S7**	Differences in overall readiness indexes by antenatal corticosteroids utilization for level 2 facilities	17
**Figure S8**	Differences in overall readiness indexes by antenatal corticosteroids utilization for level 3 facilities	18

Table S1. Sampling strategies of included SPA surveys

Survey	Sampling strategy*
Afghanistan 2018-2019	The survey focused on major public and private hospitals in the country's seven main provinces (including Nangarhar, Paktya, Kunduz, Balkh, Kandahar, and Herat, and Kabul). Among six provinces excluding Kabul, all 12 public hospitals, 37 private hospitals, and 52 private clinics were included. In Kabul, all 26 public and 20 private hospitals were surveyed, but a randomly selected sample of 13 private clinics (out of 84) were included.
Bangladesh 2017-2018	The survey adopted a stratified random sampling strategy for 1,600 facilities to obtain nationally representative data for different facility types, different facility management authorities, and each of the eight divisions (Barisal, Chittagong, Dhaka, Khulna, Mymensingh, Rajshahi, Rangpur, and Sylhet) of the country.
Nepal 2021	The survey adopted a stratified random sampling strategy for 1,633 health facilities through an equal probability systematic sampling. All government hospitals, all non-government hospitals with at least one bed, and all non-government hospitals in the provinces of Karnali and Sudurpashchim were included because of their small numbers. Also, all primary health care centers (PHCCs) and stand-alone HIV testing and counseling centers (stand-alone HTCs) were included.
Haiti 2017-2018	The survey was a national census of all operational health facilities in the country during the survey period of 2017 to 2018.
Democratic Republic of the Congo 2017-2018	The survey adopted a probability sampling strategy from 12,059 health facilities in the country, excluding health posts. On average, approximately 50 facilities were selected for each of the 26 provinces, reaching a sample of 1,380 facilities.
Ethiopia 2021-2022	The survey adopted a stratified random sampling strategy, reaching a sample of 1,407 health facilities via an unequal probability systematic sampling. Within each region, stratification was achieved by facility type. All public and private hospitals were included because of their small numbers and crucial roles in the country's health system. Health centers were sampled, but all health centers in Dire Dawa and Harari were included. Clinics were sampled with all higher clinics included, and all clinics in Harari included. Health posts were sampled.
Malawi 2013-2014	The survey was a national census of all formal-sector health facilities, including public, private, and other managing authority types, in the country during the survey period between 2013 to 2014.
Senegal 2018 and 2019	Senegal implemented a continuous SPA, which was designed to continuously collect data on health facilities over a period of 5 years from 2012 to 2017, aiming to survey all health facilities in the country.
Tanzania 2014-2015	The survey was a sample of formal-sector health facilities, including public and private, during the survey period between 2014 and 2015. The sample was selected to obtain national representative data by facility type, managing authority type, and region.

*Information obtained from SPA reports.

Table S2. The indicators used to assess facility readiness based on the 2022 WHO recommendations on ACS use

Antenatal corticosteroid therapy is recommended for women with a high likelihood of preterm birth from 24 to 34 weeks of gestation when the following conditions are met:

WHO conditions/criteria	Readiness categories	Indicators identified from the SPA survey		SPA dataset
1. Gestational age assessment can be accurately undertaken.	Readiness to accurately assess GA	Equipment	ultrasound	Inventory dataset
2. There is no clinical evidence of maternal infection.	Readiness to identify maternal infections	Equipment	thermometer	
		Diagnostics	hematology analyzer	
			HIV rapid diagnostic test	
			syphilis rapid diagnostic test	
3. Adequate childbirth care is available (including the capacity to recognize and safely manage preterm labor and birth).	Readiness to provide adequate childbirth care	Equipment	delivery pack	
			cord clamp	
			manual vacuum extractor	
			vacuum aspiration kit or D&C kit	
			forceps (large)	
			forceps (medium)	
		Medicines and commodities	parenteral antibiotics	
			parenteral anticonvulsants (diazepam)	
			parenteral oxytocin	
		Staff and guidelines	a health worker who can perform C/S	
			an anesthetist	
			national guidelines for BEmONC	
			national guidelines for CEmONC	
4. The preterm newborn can receive adequate care (including resuscitation, kangaroo mother care, thermal care, feeding support, infection treatment, and respiratory support, including continuous positive airway pressure as needed).	Readiness to provide preterm newborn care	Equipment	suction bulb or penguin sucker	
			stethoscope	
			newborn masks (0, 1), neonatal size self-inflating bag	
			incubator	
			other external heat source	
			pulse oximeter	
			oxygen concentrator	
			filled oxygen cylinder	
			oxygen distribution system	
		Medicines and commodities	glucometer	
			glucometer strips	
			hand-washing soap	
			disposable latex gloves	
		Staff and guidelines	staff trained on Integrated Management of Pregnancy and Childbirth (IMPAC)	Health worker interview dataset[1]
			staff trained in Comprehensive Emergency Obstetric and Newborn Care	
			staff trained on routine care for labor and normal vaginal delivery	
			guidelines on the management of preterm labor	Inventory dataset

*GA: gestational age; C/S Cesarean section; BEmONC: Basic Emergency Obstetric and Newborn Care; CEmONC: Comprehensive Emergency Obstetric and Newborn Care

[1] To report readiness at the facility level using data from health worker interviews, we converted data from the health-worker level to the facility level by measuring the facility with at least one health worker who had received relevant training.

Table S3. Survey response rates and number of facilities sampled, surveyed, and included in the analysis

Country	Survey year	Response rate[1]	Number of facilities sampled	Number of facilities surveyed[4]	Number (%) of facilities included in the analysis[5]
		median 94.9% (range 88.8%, 99.0%)	**N =10418** median 1130 (range 160, 1626)	**N = 9793** median 1158 (range 142, 1576)	**N = 8669** median 929 (range 108, 1500)
**South Asia**					
Afghanistan	2018-19	88.8%[2]	160	142	108 (76.1%)
Bangladesh	2017-18	95.3%	1600	1524	1498 (98.3%)
Nepal	2021	97.0%	1626	1576	1500 (95.2%)
**Caribbean**					
Haiti	2017-18	97.5%	1033	1007	929 (92.3%)
**Sub-Saharan Africa**					
DRC	2017-18	97.7%	1412	1380	1364 (98.8%)
Ethiopia	2021-22	82.3%	1407	1158	911 (78.7%)
Malawi	2013-14	92.2%	1060	977	645 (66.1%)
Senegal	2018	89.3%	466	841 (2018: 416; 2019: 425)	601 (71.5%)
Senegal	2019	94.5%[3]	454		
Tanzania	2014-15	99.0%	1200	1188	1056 (88.9%)

[1] Response rates were obtained from SPA final reports.
[2] Afghanistan 2018-2019 survey does not have a publicly available SPA final report. Its response rate was manually calculated.
[3] The response rate for Senegal 2019 is 94.5%, calculated by excluding health huts. The rate is 93.6% (425/454) if including health huts.
[4] The number of facilities surveyed is smaller than the number of facilities sampled because of non-response, refusal, or closure.
[5] Facilities included in the analysis are facilities that either provided antenatal care, performed normal deliveries, or performed Cesarean sections. The proportion was calculated by dividing the number of facilities included in the analysis by the number of facilities surveyed.

Table S4. Facility structural readiness for 35 indicators by country and facility level

Four readiness categories based on WHO criteria	The proportion of facilities with the indicators available				South Asia							
					Afghanistan 2018-19				Bangladesh 2017-18			
				Median (range)[1]	Overall	Level 1	Level 2	Level 3	Overall	Level 1	Level 2	Level 3
**1. Assess GA accurately**	**Equipment**	The proportion of facilities with a functional ultrasound machine in use		7.3% (0.2%, 12.3%)	92(82.4%)	3(41.2%)	5(67%)	84(86.2%)	178(3.6%)	13(0.7%)	19(1.7%)	92(82.4%)
**2. Identify maternal infections**	**Equipment**	The proportion of facilities with this equipment	thermometer	52.3% (20.7%, 76.2%)	65(58.6%)	0(0%)	6(75%)	59(59.4%)	734(20.7%)	0(0%)	481(85.2%)	65(58.6%)
	**Diagnostics**	The proportion of facilities with the following diagnostics	(1) hematology analyzer	7.6% (2.7%, 30.5%)	94(92%)	6(86.3%)	5(70.1%)	83(94.8%)	131(2.7%)	11(0.5%)	16(1.7%)	94(92%)
			(2) HIV rapid diagnostic test	52.4% (21.2%, 82.1%)	37(25.8%)	2(27.5%)	1(7.2%)	34(27.9%)				37(25.8%)
			(3) syphilis rapid diagnostic test	13.8% (3.0%, 68.8%)	30(21.9%)	2(27.5%)	1(7.2%)	27(23.3%)	128(3%)	18(1.1%)	22(2.7%)	30(21.9%)
**3. Provide adequate childbirth care**	**Equipment**	The proportion of facilities with the following equipment to perform CEmONC (obstetric related)	(1) delivery pack	62.5% (19.0%, 87.5%)	80(74.5%)	0(0%)	5(28.6%)	75(83.4%)	692(19%)	0(0%)	449(77.5%)	80(74.5%)
			(2) cord clamp	55.8% (15.8%, 82.1%)	78(70.3%)	0(0%)	6(37.3%)	72(77.5%)	566(15.8%)	0(0%)	358(63.6%)	78(70.3%)
			(3) manual vacuum extractor	9.1% (4.7%, 33.9%)	76(69.1%)	0(0%)	3(17.4%)	73(78.4%)	214(4.8%)	0(0%)	90(14.7%)	76(69.1%)
			(4) vacuum aspiration kit or D&C kit	10.9% (5.8%, 49.4%)	74(60.5%)	0(0%)	6(37%)	68(66.1%)	303(5.8%)	0(0%)	125(14.5%)	74(60.5%)
			(5) forceps (large)	32.2% (5.4%, 73.5%)	85(74.9%)	0(0%)	10(100%)	75(75.6%)	718(19.7%)	0(0%)	473(80.4%)	85(74.9%)
			(6) forceps (medium)	34.8% (5.2%, 82.2%)	84(74.3%)	0(0%)	10(100%)	74(74.8%)	697(19.4%)	0(0%)	451(79.4%)	84(74.3%)
	**Medicines and commodities**	The proportion of facilities with the following medicines	(1) parenteral antibiotics	31.4% (4.6%, 51.3%)	45(42%)	0(0%)	2(50%)	43(43%)	281(4.6%)	0(0%)	82(7.6%)	45(42%)
			(2) parenteral anticonvulsants (diazepam)	31.8% (4.7%, 66.6%)	49(45.1%)	0(0%)	2(50%)	47(46.7%)	264(4.7%)	0(0%)	73(8.6%)	49(45.1%)
			(3) parenteral oxytocin	56.4% (7.5%, 79.2%)	78(74.4%)	0(0%)	5(32.2%)	73(82.8%)	401(7.5%)	0(0%)	178(21.6%)	78(74.4%)
	**Staff and guidelines**	The proportion of facilities that has the following staff	(1) a health worker who can perform C/S	4.0% (2.2%, 20.5%)	46(32.5%)	0(0%)	0(0%)	46(37.8%)	158(2.5%)	0(0%)	0(0%)	46(32.5%)
			(2) an anesthetist	3.8% (1.9%, 12.3%)	36(26.6%)	0(0%)	0(0%)	36(31%)	118(1.9%)	0(0%)	0(0%)	36(26.6%)
		The proportion of facilities with the following guidelines	(1) national guidelines for BEmONC	18.2% (2.8%, 40.9%)	9(5.1%)	0(0%)	0(0%)	9(5.9%)	132(2.8%)	0(0%)	80(10.9%)	9(5.1%)
			(2) national guidelines for CEmONC	13.4% (2.2%, 41.9%)	9(5.1%)	0(0%)	0(0%)	9(5.9%)	114(2.2%)	0(0%)	65(8.7%)	9(5.1%)
**4. Provide adequate preterm newborn care**	**Equipment**	The proportion of facilities with the following equipment for neonatal resuscitation	(1) suction bulb or penguin sucker	46.2% (15.8%, 80.4%)	78(81.6%)	0(0%)	7(79.4%)	71(85.7%)	652(15.8%)	0(0%)	414(62.6%)	78(81.6%)
			(2) stethoscope (in general)	51.1% (23.1%, 81.3%)	87(87.4%)	0(0%)	5(69.7%)	82(93.7%)	804(23.2%)	0(0%)	540(97.6%)	87(87.4%)
			(3) newborn masks, neonatal size self-inflating bag	37.3% (13.1%, 74.4%)	83(77.3%)	0(0%)	6(37%)	77(85.7%)	601(13.1%)	0(0%)	358(48%)	83(77.3%)
		The proportion of facilities with equipment for thermal care	(1) incubator	4.5% (2.2%, 6.6%)	54(55.4%)	0(0%)	2(11.1%)	52(63.3%)	127(2.2%)	0(0%)	29(2.7%)	54(55.4%)
			(2) other external heat source	14.6% (4.2%, 58.4%)	68(64.1%)	0(0%)	3(16.7%)	65(72.7%)	176(4.2%)	0(0%)	61(10.7%)	68(64.1%)
		The proportion of facilities with equipment for respiratory care, including safe oxygen use	(1) pulse oximeter	8.8% (2.8%, 42.0%)	49(53.7%)	1(13.7%)	1(5.6%)	47(61.2%)	142(3%)	7(0.4%)	18(1.1%)	49(53.7%)
			(2) oxygen concentrator	9.2% (2.4%, 15.2%)	40(48.2%)	0(0%)	0(0%)	40(56.1%)	192(3.2%)	6(0.3%)	69(5.8%)	40(48.2%)
			(3) filled oxygen cylinder	9.8% (1.8%, 22.5%)	82(84.9%)	3(45.1%)	7(82.4%)	72(87.1%)	334(6.6%)	23(1%)	122(13.2%)	82(84.9%)
			(4) oxygen distribution system	3.5% (1.6%, 8.5%)	16(13.3%)	2(27.5%)	1(5.6%)	13(13.5%)	84(1.6%)	2(0.1%)	26(2%)	16(13.3%)
	**Medicines and commodities**	The proportion of facilities with commodities for monitoring blood glucose	(1) glucometer	22.2% (19.3%, 79.7%)	42(37%)	0(0%)	3(55.5%)	39(36.6%)	310(21.1%)	124(21.7%)	64(10.6%)	42(37%)
			(2) glucometer strips	20.6% (17.4%, 82.0%)	37(34%)	0(0%)	3(55.5%)	34(33.1%)	303(21.3%)	126(22.2%)	57(9.9%)	37(34%)
		The proportion of facilities with medicines and commodities for infection management	(1) hand-washing soap	50.8% (45.3%, 88.5%)	71(62.6%)	7(100%)	4(22.7%)	60(65.5%)	1075(72.8%)	473(72.1%)	406(77.3%)	71(62.6%)
			(2) disposable latex gloves	89.8% (69.4%, 96.4%)	102(88.9%)	7(100%)	8(50.5%)	87(92.9%)	1158(73.7%)	458(70.3%)	472(84.7%)	102(88.9%)
	**Staff and guidelines**	The proportion of facilities with at least one health worker who has received training about the following in the past 24 months	(1) Integrated Management of Pregnancy and Childbirth (IMPAC)	18.6% (2.4%, 37.8%)	15(12.9%)	1(13.7%)	0(0%)	14(14.3%)	116(15%)	32(16.6%)	45(11.7%)	15(12.9%)
			(2) Comprehensive Emergency Obstetric and Newborn Care (CEmONC)	17.6% (2.0%, 29.6%)	7(4.2%)	0(0%)	0(0%)	7(4.9%)	84(10.6%)	19(12.3%)	32(6.1%)	7(4.2%)
			(3) Routine care for labor and normal vaginal delivery	24.0% (2.8%, 43.5%)	16(13.5%)	1(13.7%)	0(0%)	15(15.1%)	153(18.2%)	32(20.5%)	78(14.8%)	16(13.5%)
		The proportion of facilities with the following guidelines	guidelines on management of preterm labor	10.2% (3.2%, 34.7%)	9(5.1%)	0(0%)	0(0%)	9(5.9%)	140(3.2%)	0(0%)	85(13.6%)	9(5.1%)

[1] Median and range were obtained from data across eight countries (excluding Afghanistan).
* (grey box) : data unavailable

Table S4. Facility structural readiness for 35 indicators by country and facility level (continued)

Four readiness categories based on WHO criteria	The proportion of facilities with the indicators available			South Asia Nepal 2021				Caribbean Haiti 2017-2018			
				Overall	Level 1	Level 2	Level 3	Overall	Level 1	Level 2	Level 3
**1. Assess GA accurately**	**Equipment**	The proportion of facilities with a functional ultrasound machine in use		422(12.3%)	61(5.3%)	135(10.3%)	226(93.3%)	100(10.8%)	18(3.2%)	14(5.5%)	68(63.5%)
**2. Identify maternal infections**	**Equipment**	The proportion of facilities with this equipment	thermometer	731(48%)	0(0%)	515(91.7%)	216(90.1%)	310(33.4%)	0(0%)	227(89%)	83(77.5%)
	**Diagnostics**	The proportion of facilities with the following diagnostics	(1) hematology analyzer	309(8.6%)	37(3.8%)	70(5.4%)	202(79.3%)	283(30.5%)	123(21.7%)	88(34.5%)	72(67.3%)
			(2) HIV rapid diagnostic test					197(21.2%)	97(17.1%)	68(26.6%)	32(29.9%)
			(3) syphilis rapid diagnostic test	304(12.8%)	69(7.2%)	149(16.2%)	86(32.4%)	194(20.9%)	106(18.7%)	60(23.5%)	28(26.2%)
**3. Provide adequate childbirth care**	**Equipment**	The proportion of facilities with the following equipment to perform CEmONC (obstetric related)	(1) delivery pack	767(50.6%)	0(0%)	549(97.2%)	218(88.6%)	283(30.5%)	0(0%)	198(77.6%)	85(79.4%)
			(2) cord clamp	714(46.8%)	0(0%)	505(89.4%)	209(87.1%)	314(33.8%)	0(0%)	218(85.5%)	96(89.6%)
			(3) manual vacuum extractor	313(12.1%)	0(0%)	137(17.6%)	176(70.8%)	51(5.5%)	0(0%)	20(7.8%)	31(28.9%)
			(4) vacuum aspiration kit or D&C kit	326(10.9%)	0(0%)	138(14.5%)	188(76.6%)	100(10.8%)	0(0%)	55(21.6%)	45(42%)
			(5) forceps (large)	640(40.6%)	0(0%)	441(76.7%)	199(83.9%)	50(5.4%)	0(0%)	17(6.7%)	33(30.8%)
			(6) forceps (medium)	707(46.1%)	0(0%)	499(88%)	208(87.1%)	49(5.3%)	0(0%)	20(7.9%)	29(27.1%)
	**Medicines and commodities**	The proportion of facilities with the following medicines	(1) parenteral antibiotics	575(34.4%)	0(0%)	388(64%)	187(79%)	179(19.3%)	0(0%)	109(42.8%)	70(65.3%)
			(2) parenteral anticonvulsants (diazepam)	358(14.7%)	0(0%)	175(22.4%)	183(76.8%)	112(12.1%)	0(0%)	67(26.3%)	45(41.9%)
			(3) parenteral oxytocin	760(50.5%)	0(0%)	547(97.4%)	213(86.7%)	257(27.7%)	0(0%)	173(67.9%)	84(78.4%)
	**Staff and guidelines**	The proportion of facilities that has the following staff	(1) a health worker who can perform C/S	166(3.5%)	0(0%)	0(0%)	166(65.6%)	54(5.8%)	0(0%)	0(0%)	54(50.4%)
			(2) an anesthetist	152(3.2%)	0(0%)	0(0%)	152(60.4%)	43(4.6%)	0(0%)	0(0%)	43(40.1%)
		The proportion of facilities with the following guidelines	(1) national guidelines for BEmONC								
			(2) national guidelines for CEmONC					124(13.4%)	0(0%)	88(34.5%)	36(33.6%)
**4. Provide adequate preterm newborn care**	**Equipment**	The proportion of facilities with the following equipment for neonatal resuscitation	(1) suction bulb or penguin sucker	393(22.3%)	0(0%)	252(40.4%)	141(61.2%)	325(35%)	0(0%)	226(88.6%)	99(92.5%)
			(2) stethoscope (in general)	771(51.1%)	0(0%)	551(98%)	220(91.9%)	335(36.1%)	0(0%)	242(94.9%)	93(86.8%)
			(3) newborn masks, neonatal size self-inflating bag	718(47.7%)	0(0%)	511(91.5%)	207(86.2%)	207(22.3%)	0(0%)	122(47.8%)	85(79.5%)
		The proportion of facilities with equipment for thermal care	(1) incubator	141(4.5%)	0(0%)	42(4.7%)	99(43.6%)	59(6.4%)	0(0%)	24(9.5%)	35(32.7%)
			(2) other external heat source	530(33.6%)	0(0%)	354(63.2%)	176(72.8%)	160(17.2%)	0(0%)	90(35.3%)	70(65.4%)
		The proportion of facilities with equipment for respiratory care, including safe oxygen use	(1) pulse oximeter	770(42%)	211(28.1%)	340(50.4%)	219(91.8%)	155(16.7%)	58(10.2%)	54(21.2%)	43(40.1%)
			(2) oxygen concentrator	307(11.7%)	37(4.1%)	145(14.7%)	125(52.3%)	100(10.8%)	23(4.1%)	40(15.7%)	37(34.6%)
			(3) filled oxygen cylinder	507(22.5%)	85(8.5%)	237(30.3%)	185(78.7%)	149(16.1%)	36(6.3%)	55(21.7%)	58(54.2%)
			(4) oxygen distribution system	128(3.2%)	11(1%)	7(0.6%)	110(45.4%)	32(3.5%)	5(0.9%)	11(4.4%)	16(14.9%)
	**Medicines and commodities**	The proportion of facilities with commodities for monitoring blood glucose	(1) glucometer	348(19.4%)	94(11.4%)	150(24.9%)	104(42.6%)	463(49.8%)	227(40%)	159(62.3%)	77(71.9%)
			(2) glucometer strips	325(17.4%)	82(10%)	138(21.9%)	105(42.9%)	424(45.6%)	201(35.4%)	146(57.2%)	77(71.9%)
		The proportion of facilities with medicines and commodities for infection management	(1) hand-washing soap	1297(88.5%)	595(86.2%)	507(91.7%)	195(81.8%)	492(52.9%)	305(53.8%)	133(52.1%)	54(50.5%)
			(2) disposable latex gloves	1433(95.8%)	651(93.8%)	554(97.9%)	228(94.2%)	831(89.5%)	497(87.7%)	235(92.2%)	99(92.5%)
	**Staff and guidelines**	The proportion of facilities with at least one health worker who has received training about the following in the past 24 months	(1) Integrated Management of Pregnancy and Childbirth (IMPAC)	87(4.5%)	4(0.7%)	54(7.7%)	29(10.2%)	257(27.8%)	76(13.5%)	109(42.8%)	72(67.3%)
			(2) Comprehensive Emergency Obstetric and Newborn Care (CEmONC)	98(4.4%)	5(1.2%)	57(6.8%)	36(12.5%)	218(23.5%)	59(10.4%)	91(35.7%)	68(63.5%)
			(3) Routine care for labor and normal vaginal delivery	180(11.1%)	7(1%)	119(20.4%)	54(18.9%)	258(27.9%)	75(13.3%)	111(43.6%)	72(67.3%)
		The proportion of facilities with the following guidelines	guidelines on management of preterm labor					60(6.4%)	0(0%)	40(15.6%)	20(18.6%)

* ░ : data unavailable

Table S4. Facility structural readiness for 35 indicators by country and facility level (continued)

Four readiness categories based on WHO criteria	The proportion of facilities with the indicators available			Sub-Saharan Africa							
				DRC 2017-2018				Ethiopia 2021-2022			
				Overall	Level 1	Level 2	Level 3	Overall	Level 1	Level 2	Level 3
**1. Assess GA accurately**	**Equipment**	The proportion of facilities with a functional ultrasound machine in use		450(10.7%)	1(12.5%)	8(1.2%)	441(36.2%)	344(4.5%)	16(1.2%)	38(6.1%)	290(84.9%)
**2. Identify maternal infections**	**Equipment**	The proportion of facilities with this equipment	thermometer	1102(76.2%)	0(0%)	377(74.6%)	725(85.9%)	568(21%)	0(0%)	262(83.5%)	306(91%)
	**Diagnostics**	The proportion of facilities with the following diagnostics	(1) hematology analyzer	179(5.4%)	1(12.5%)	8(2%)	170(14.1%)	364(5.7%)	17(1%)	68(12.4%)	279(82.2%)
			(2) HIV rapid diagnostic test	654(44.2%)	4(28.1%)	189(39.3%)	461(58.5%)	616(29.1%)	62(10.5%)	258(85.6%)	296(80.1%)
			(3) syphilis rapid diagnostic test	218(11.8%)	1(12.5%)	53(9.9%)	164(16.9%)	238(11.1%)	24(5.2%)	107(29.1%)	107(28.2%)
**3. Provide adequate childbirth care**	**Equipment**	The proportion of facilities with the following equipment to perform CEmONC (obstetric related)	(1) delivery pack	1240(87.5%)	0(0%)	437(87.2%)	803(94.5%)	605(22.5%)	0(0%)	276(89.5%)	329(96.9%)
			(2) cord clamp	1160(79.1%)	0(0%)	409(80.1%)	751(81.9%)	625(23.4%)	0(0%)	292(93.4%)	333(97.9%)
			(3) manual vacuum extractor	273(7.2%)	0(0%)	28(5.1%)	245(13.4%)	518(16.5%)	0(0%)	205(62.8%)	313(92.8%)
			(4) vacuum aspiration kit or D&C kit	729(36.4%)	0(0%)	134(26.4%)	595(65.9%)	385(10%)	0(0%)	124(36.2%)	261(70.4%)
			(5) forceps (large)	127(5.9%)	0(0%)	31(5.4%)	96(7.6%)	617(23.9%)	0(0%)	286(95.4%)	331(97.4%)
			(6) forceps (medium)	112(5.2%)	0(0%)	21(4.6%)	91(7.1%)	611(23.4%)	0(0%)	281(93.4%)	330(96.4%)
	**Medicines and commodities**	The proportion of facilities with the following medicines	(1) parenteral antibiotics	549(36.4%)	0(0%)	174(34.3%)	375(44.4%)	489(16.4%)	0(0%)	204(64.6%)	285(75.7%)
			(2) parenteral anticonvulsants (diazepam)	785(55.4%)	0(0%)	253(52.7%)	532(66.6%)	351(10.4%)	0(0%)	121(39.3%)	230(61.6%)
			(3) parenteral oxytocin	1110(77.3%)	0(0%)	394(77.5%)	716(82.4%)	611(22.8%)	0(0%)	283(90.7%)	328(96.7%)
	**Staff and guidelines**	The proportion of facilities that has the following staff	(1) a health worker who can perform C/S	725(20.5%)	0(0%)	0(0%)	725(77.3%)	312(2.2%)	0(0%)	0(0%)	312(83.5%)
			(2) an anesthetist	486(12.3%)	0(0%)	0(0%)	486(46.4%)	311(2.2%)	0(0%)	0(0%)	311(82.8%)
		The proportion of facilities with the following guidelines	(1) national guidelines for BEmONC					350(11.5%)	0(0%)	147(45%)	203(55%)
			(2) national guidelines for CEmONC	411(24.7%)	0(0%)	118(23.2%)	293(30.5%)	162(4.3%)	0(0%)	45(15.5%)	117(33%)
**4. Provide adequate preterm newborn care**	**Equipment**	The proportion of facilities with the following equipment for neonatal resuscitation	(1) suction bulb or penguin sucker	1099(80.4%)	0(0%)	388(80%)	711(87.1%)	577(21.3%)	0(0%)	261(84.3%)	316(93.6%)
			(2) stethoscope (in general)	1144(81.3%)	0(0%)	412(81.5%)	732(86.3%)	612(23.1%)	0(0%)	290(92.1%)	322(95.1%)
			(3) newborn masks, neonatal size self-inflating bag	561(26.9%)	0(0%)	102(21%)	459(44.6%)	593(21.6%)	0(0%)	262(85.2%)	331(97.4%)
		The proportion of facilities with equipment for thermal care	(1) incubator	114(3%)	0(0%)	3(0.6%)	111(9.7%)	232(4.5%)	0(0%)	60(14.4%)	172(47.1%)
			(2) other external heat source	411(18.2%)	0(0%)	62(12.4%)	349(35.3%)	382(9.4%)	0(0%)	118(32.7%)	264(79.5%)
		The proportion of facilities with equipment for respiratory care, including safe oxygen use	(1) pulse oximeter	78(2.8%)	1(12.5%)	2(0.4%)	75(8.6%)	318(26.8%)	23(31.1%)	85(21%)	210(64.9%)
			(2) oxygen concentrator	105(2.4%)	2(14.4%)	2(0.5%)	101(6.7%)	184(11.2%)	11(11.3%)	38(6.8%)	135(46.5%)
			(3) filled oxygen cylinder	73(1.8%)	2(14.4%)	1(0.3%)	70(4.8%)	200(13%)	12(15.7%)	43(7.9%)	145(49%)
			(4) oxygen distribution system					80(4.8%)	3(7.2%)	12(2.6%)	65(17.7%)
	**Medicines and commodities**	The proportion of facilities with commodities for monitoring blood glucose	(1) glucometer	761(34%)	3(27.7%)	76(18.2%)	682(77%)	564(21%)	38(4.1%)	202(68.8%)	324(95.6%)
			(2) glucometer strips	674(29.6%)	3(27.7%)	63(15.6%)	608(67.4%)	542(19.3%)	35(3.8%)	188(62.5%)	319(94.4%)
		The proportion of facilities with medicines and commodities for infection management	(1) hand-washing soap	686(48.4%)	7(57.8%)	228(45.6%)	451(55.6%)	487(45.3%)	116(42.7%)	160(51.8%)	211(63.3%)
			(2) disposable latex gloves	1256(90.2%)	10(100%)	453(88.5%)	793(93.9%)	761(69.4%)	160(61.5%)	283(93%)	318(94.1%)
	**Staff and guidelines**	The proportion of facilities with at least one health worker who has received training about the following in the past 24 months	(1) Integrated Management of Pregnancy and Childbirth (IMPAC)	423(34.5%)	1(41.7%)	107(33.8%)	315(35.6%)	108(2.4%)	5(0.4%)	41(6.6%)	62(24.7%)
			(2) Comprehensive Emergency Obstetric and Newborn Care (CEmONC)	401(29.6%)	1(41.7%)	93(28.6%)	307(31.2%)	112(2%)	4(0.3%)	38(5.6%)	70(19.3%)
			(3) Routine care for labor and normal vaginal delivery	415(33.6%)	1(41.7%)	107(33.5%)	307(33.8%)	126(2.8%)	7(1.1%)	44(6.5%)	75(20.6%)
		The proportion of facilities with the following guidelines	guidelines on management of preterm labor	232(11.4%)	0(0%)	46(8.8%)	186(19%)	248(7.2%)	0(0%)	86(27.1%)	162(43.2%)

* (grey cell) : data unavailable

Table S4. Facility structural readiness for 35 indicators by country and facility level (continued)

Four readiness categories based on WHO criteria	The proportion of facilities with the indicators available			Sub-Saharan Africa							
				Malawi 2013-2014				Senegal 2018 and 2019			
				Overall	Level 1	Level 2	Level 3	Overall	Level 1	Level 2	Level 3
**1. Assess GA accurately**	**Equipment**	The proportion of facilities with a functional ultrasound machine in use		65(10%)	4(3.8%)	7(1.5%)	54(76%)	1(0.2%)	0(0%)	1(0.2%)	0(0%)
**2. Identify maternal infections**	**Equipment**	The proportion of facilities with this equipment	thermometer	453(69.6%)	0(0%)	390(82.6%)	63(88.6%)	495(69.9%)	0(0%)	436(80.2%)	59(96.4%)
	**Diagnostics**	The proportion of facilities with the following diagnostics	(1) hematology analyzer	57(8.8%)	8(7.7%)	6(1.3%)	43(60.8%)	139(16.1%)	7(28.9%)	80(10.5%)	52(52.2%)
			(2) HIV rapid diagnostic test	391(60.6%)	57(55.4%)	288(61.2%)	46(64.6%)	548(78.7%)	40(40.5%)	461(86.2%)	47(72.7%)
			(3) syphilis rapid diagnostic test	96(14.8%)	17(16.4%)	54(11.4%)	25(35.2%)	487(68.8%)	37(39.5%)	410(77.6%)	40(30.2%)
**3. Provide adequate childbirth care**	**Equipment**	The proportion of facilities with the following equipment to perform CEmONC (obstetric related)	(1) delivery pack	486(74.8%)	0(0%)	418(88.7%)	68(95.7%)	579(83.7%)	0(0%)	521(97.8%)	58(96%)
			(2) cord clamp	482(74.1%)	0(0%)	416(88.2%)	66(92.9%)	568(82.1%)	0(0%)	509(95.8%)	59(96.4%)
			(3) manual vacuum extractor	221(33.9%)	0(0%)	165(34.8%)	56(78.8%)	96(11%)	0(0%)	57(7.6%)	39(71.5%)
			(4) vacuum aspiration kit or D&C kit	131(20.1%)	0(0%)	97(20.4%)	34(48%)	343(49.4%)	0(0%)	303(55.7%)	40(79.1%)
			(5) forceps (large)	448(69%)	0(0%)	390(82.8%)	58(81.7%)	521(73.5%)	0(0%)	464(85%)	57(94.9%)
			(6) forceps (medium)	434(66.9%)	0(0%)	379(80.5%)	55(77.5%)	533(75.6%)	0(0%)	474(87.5%)	59(96.4%)
	**Medicines and commodities**	The proportion of facilities with the following medicines	(1) parenteral antibiotics	299(46.1%)	0(0%)	244(51.9%)	55(77.4%)	362(51.3%)	0(0%)	316(60.7%)	46(51.1%)
			(2) parenteral anticonvulsants (diazepam)	433(66.6%)	0(0%)	371(78.6%)	62(87.2%)	363(51.6%)	0(0%)	317(58.1%)	46(83.6%)
			(3) parenteral oxytocin	514(79.2%)	0(0%)	449(95.3%)	65(91.4%)	451(62.3%)	0(0%)	402(74.5%)	49(52.2%)
	**Staff and guidelines**	The proportion of facilities that has the following staff	(1) a health worker who can perform C/S	57(8.7%)	0(0%)	0(0%)	57(80.1%)	49(3.5%)	0(0%)	0(0%)	49(48.7%)
			(2) an anesthetist	47(7.2%)	0(0%)	0(0%)	47(66%)	51(3.6%)	0(0%)	0(0%)	51(50.4%)
		The proportion of facilities with the following guidelines	(1) national guidelines for BEmONC	266(40.9%)	0(0%)	231(48.9%)	35(49.1%)				
			(2) national guidelines for CEmONC	148(22.8%)	0(0%)	117(24.8%)	31(43.6%)	304(41.9%)	0(0%)	269(47.5%)	35(64.5%)
**4. Provide adequate preterm newborn care**	**Equipment**	The proportion of facilities with the following equipment for neonatal resuscitation	(1) suction bulb or penguin sucker	399(61.5%)	0(0%)	346(73.6%)	53(74.5%)	480(66.4%)	0(0%)	434(76.9%)	46(84.9%)
			(2) stethoscope (in general)	423(65.2%)	0(0%)	369(78.4%)	54(76.1%)	369(51.1%)	0(0%)	314(56.7%)	55(92.2%)
			(3) newborn masks, neonatal size self-inflating bag	483(74.4%)	0(0%)	418(88.7%)	65(91.4%)	477(67.4%)	0(0%)	419(77.1%)	58(94.9%)
		The proportion of facilities with equipment for thermal care	(1) incubator	37(5.7%)	0(0%)	8(1.7%)	29(41.1%)	41(6.6%)	0(0%)	18(3%)	23(59.4%)
			(2) other external heat source	78(12%)	0(0%)	31(6.5%)	47(66.3%)	407(58.4%)	0(0%)	350(65.9%)	57(94.2%)
		The proportion of facilities with equipment for respiratory care, including safe oxygen use	(1) pulse oximeter	60(9.3%)	9(8.8%)	25(5.3%)	26(36.9%)	68(8.2%)	2(1.2%)	22(2.5%)	44(85.1%)
			(2) oxygen concentrator	99(15.2%)	9(8.5%)	54(11.4%)	36(50.7%)	61(7.5%)	1(0.6%)	23(2.9%)	37(73.4%)
			(3) filled oxygen cylinder	47(7.3%)	7(6.8%)	16(3.4%)	24(34%)	99(12.4%)	5(10.9%)	44(6%)	50(85.7%)
			(4) oxygen distribution system	27(4.2%)	2(1.9%)	15(3.2%)	10(14.2%)	60(8.5%)	1(9.1%)	16(2.1%)	43(78.4%)
	**Medicines and commodities**	The proportion of facilities with commodities for monitoring blood glucose	(1) glucometer	151(23.4%)	32(31.2%)	58(12.3%)	61(85.8%)	532(79.7%)	48(72.9%)	446(81.1%)	38(77.7%)
			(2) glucometer strips	129(20%)	24(23.4%)	48(10.1%)	57(80.2%)	537(82%)	46(71%)	446(84%)	45(82.5%)
		The proportion of facilities with medicines and commodities for infection management	(1) hand-washing soap	313(48.7%)	57(55.4%)	211(44.9%)	45(63.5%)	334(48.2%)	36(44.9%)	263(50.1%)	35(34.6%)
			(2) disposable latex gloves	622(96.4%)	96(93%)	458(97.2%)	68(95.8%)	584(86.7%)	55(81.7%)	480(91.5%)	49(44.4%)
	**Staff and guidelines**	The proportion of facilities with at least one health worker who has received training about the following in the past 24 months	(1) Integrated Management of Pregnancy and Childbirth (IMPAC)	137(21.2%)	3(2.9%)	103(21.9%)	31(44.2%)	223(37.8%)	10(19%)	190(41.8%)	23(21.3%)
			(2) Comprehensive Emergency Obstetric and Newborn Care (CEmONC)	133(20.6%)	5(4.8%)	97(20.6%)	31(44.3%)	170(27.5%)	9(17.5%)	141(29.6%)	20(18.6%)
			(3) Routine care for labor and normal vaginal delivery	214(33.2%)	3(2.9%)	166(35.3%)	45(64.1%)	236(43.5%)	10(46.1%)	205(45.4%)	21(18.8%)
		The proportion of facilities with the following guidelines	guidelines on management of preterm labor	226(34.7%)	0(0%)	190(40.2%)	36(50.6%)	130(18%)	0(0%)	107(17.7%)	23(57.6%)

* (grey cell) : data unavailable

Table S4. Facility structural readiness for 35 indicators by country and facility level (continued)

Four readiness categories based on WHO criteria		The proportion of facilities with the indicators available		Sub-Saharan Africa<br>Tanzania 2014-2015<br>Overall	Level 1	Level 2	Level 3
**1. Assess GA accurately**	**Equipment**	The proportion of facilities with a functional ultrasound machine in use		223(4.6%)	6(2.9%)	23(1%)	194(65.1%)
**2. Identify maternal infections**	**Equipment**	The proportion of facilities with this equipment	thermometer	654(56.6%)	0(0%)	417(62.7%)	237(84.6%)
	**Diagnostics**	The proportion of facilities with the following diagnostics	(1) hematology analyzer	233(6.7%)	12(10.1%)	52(3%)	169(58.2%)
			(2) HIV rapid diagnostic test	879(82.1%)	76(77.4%)	573(82.6%)	230(83%)
			(3) syphilis rapid diagnostic test	455(40%)	50(41.1%)	275(39.5%)	130(45.4%)
**3. Provide adequate childbirth care**	**Equipment**	The proportion of facilities with the following equipment to perform CEmONC (obstetric related)	(1) delivery pack	838(74.4%)	0(0%)	575(83.2%)	263(97.1%)
			(2) cord clamp	711(64.7%)	0(0%)	487(72.4%)	224(83.3%)
			(3) manual vacuum extractor	176(4.7%)	0(0%)	26(2.2%)	150(54.5%)
			(4) vacuum aspiration kit or D&C kit	197(6.6%)	0(0%)	77(5.1%)	120(45.7%)
			(5) forceps (large)	821(72.1%)	0(0%)	568(80.7%)	253(93.3%)
			(6) forceps (medium)	896(82.2%)	0(0%)	634(92.5%)	262(96.9%)
	**Medicines and commodities**	The proportion of facilities with the following medicines	(1) parenteral antibiotics	363(28.4%)	0(0%)	211(30.7%)	152(54.4%)
			(2) parenteral anticonvulsants (diazepam)	580(48.9%)	0(0%)	369(53.9%)	211(76.7%)
			(3) parenteral oxytocin	818(69.8%)	0(0%)	562(77.9%)	256(93.2%)
	**Staff and guidelines**	The proportion of facilities that has the following staff	(1) a health worker who can perform C/S	238(4.4%)	0(0%)	0(0%)	238(83.6%)
			(2) an anesthetist	215(3.9%)	0(0%)	0(0%)	215(74.3%)
		The proportion of facilities with the following guidelines	(1) national guidelines for BEmONC	368(25%)	0(0%)	249(27.3%)	119(43.2%)
			(2) national guidelines for CEmONC	134(7.9%)	0(0%)	79(8.1%)	55(20.7%)
**4. Provide adequate preterm newborn care**	**Equipment**	The proportion of facilities with the following equipment for neonatal resuscitation	(1) suction bulb or penguin sucker	652(57.4%)	0(0%)	427(63.9%)	225(80.7%)
			(2) stethoscope (in general)	732(64%)	0(0%)	490(71.4%)	242(86.4%)
			(3) newborn masks, neonatal size self-inflating bag	772(67.4%)	0(0%)	512(75%)	260(94.8%)
		The proportion of facilities with equipment for thermal care	(1) incubator	97(2.4%)	0(0%)	17(1.1%)	80(28%)
			(2) other external heat source	188(5.6%)	0(0%)	41(3.4%)	147(52.5%)
		The proportion of facilities with equipment for respiratory care, including safe oxygen use	(1) pulse oximeter	84(2.8%)	8(10.1%)	9(0.4%)	67(25.3%)
			(2) oxygen concentrator	159(5.2%)	11(12.2%)	45(2.1%)	103(38.7%)
			(3) filled oxygen cylinder	105(3.7%)	8(11.1%)	20(1%)	77(29.7%)
			(4) oxygen distribution system	51(2.1%)	5(5.5%)	12(0.9%)	34(12.9%)
	**Medicines and commodities**	The proportion of facilities with commodities for monitoring blood glucose	(1) glucometer	453(19.3%)	40(42.2%)	181(12%)	232(86%)
			(2) glucometer strips	395(17.8%)	35(39.3%)	147(11%)	213(78.4%)
		The proportion of facilities with medicines and commodities for infection management	(1) hand-washing soap	697(63.5%)	67(64.1%)	434(63.1%)	196(70%)
			(2) disposable latex gloves	996(94%)	98(94.1%)	640(94%)	258(94.9%)
	**Staff and guidelines**	The proportion of facilities with at least one health worker who has received training about the following in the past 24 months	(1) Integrated Management of Pregnancy and Childbirth (IMPAC)	228(16%)	3(5.1%)	138(16.6%)	87(31.5%)
			(2) Comprehensive Emergency Obstetric and Newborn Care (CEmONC)	252(14.7%)	2(5%)	146(14.6%)	104(36.8%)
			(3) Routine care for labor and normal vaginal delivery	308(20.1%)	6(9.2%)	185(20.2%)	117(42.6%)
		The proportion of facilities with the following guidelines	guidelines on management of preterm labor	153(10.2%)	0(0%)	83(10.7%)	70(24.9%)

* : data unavailable

Figure S1. ACS utilization and corticosteroid availability by facility level

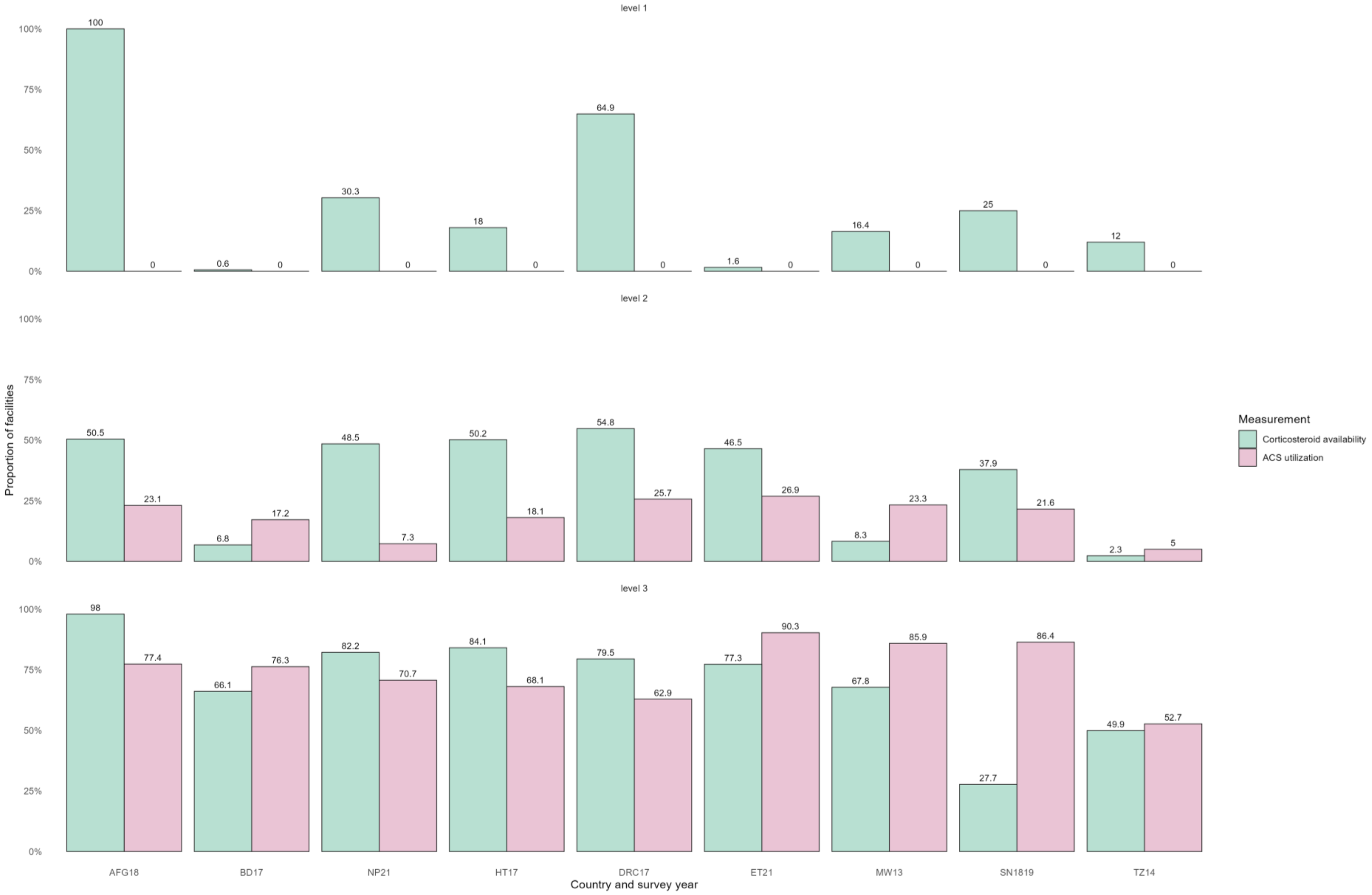

AFG18: Afghanistan 2018-2019; BD17: Bangladesh 2017-2018; NP21: Nepal 2021; HT17: Haiti 2017-2018; DRC17: Democratic Republic of the Congo 2017-2018; ET21: Ethiopia 2021-2022; MW13: Malawi 2013-2014; SN1819: Senegal 2018 and 2019; TZ14: Tanzania 2014-2015

Figure S2. Heatmap of facility readiness for all facilities by country

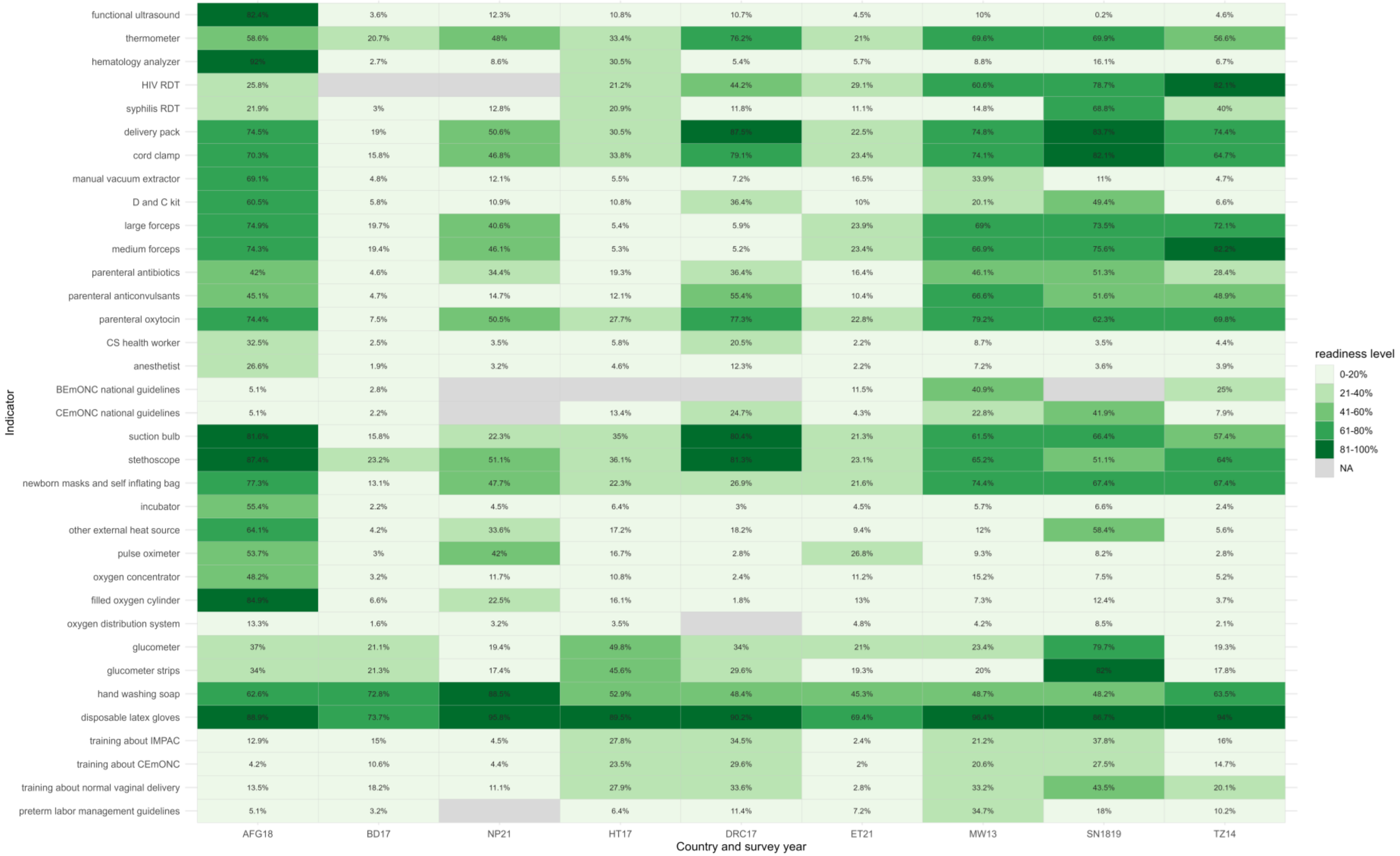

AFG18: Afghanistan 2018-2019; BD17: Bangladesh 2017-2018; NP21: Nepal 2021; HT17: Haiti 2017; DRC17: Democratic Republic of the Congo 2017-2018; ET21: Ethiopia 20121-2022; MW13: Malawi 2013-2014; SN1819: Senegal 2018 and 2019; TZ14: Tanzania 2014-2015;

RDT: rapid diagnostic test; BEmONC: Basic Emergency Obstetrics and Newborn Care; CEmONC: Comprehensive Emergency Obstetrics and Newborn Care; IMPAC: Integrated Management of Pregnancy and Childbirth; NA: not applicable

Figure S3. Heatmap of facility readiness for level 1 facilities by country

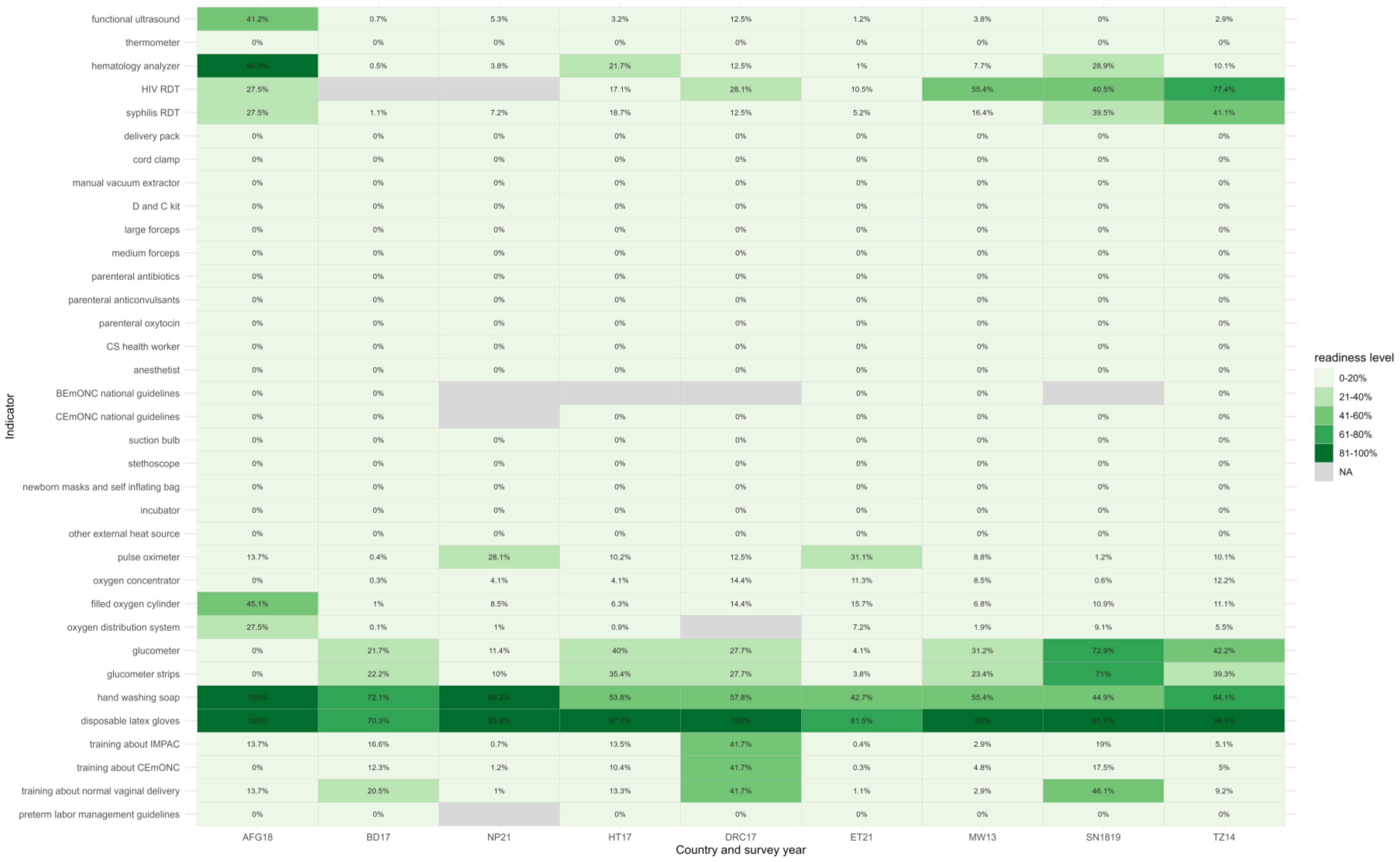

AFG18: Afghanistan 2018-2019; BD17: Bangladesh 2017-2018; NP21: Nepal 2021; HT17: Haiti 2017; DRC17: Democratic Republic of the Congo 2017-2018; ET21: Ethiopia 20121-2022; MW13: Malawi 2013-2014; SN1819: Senegal 2018 and 2019; TZ14: Tanzania 2014-2015;

RDT: rapid diagnostic test; BEmONC: Basic Emergency Obstetrics and Newborn Care; CEmONC: Comprehensive Emergency Obstetrics and Newborn Care; IMPAC: Integrated Management of Pregnancy and Childbirth; NA: not applicable

Figure S4. Heatmap of facility readiness for level 2 facilities by country

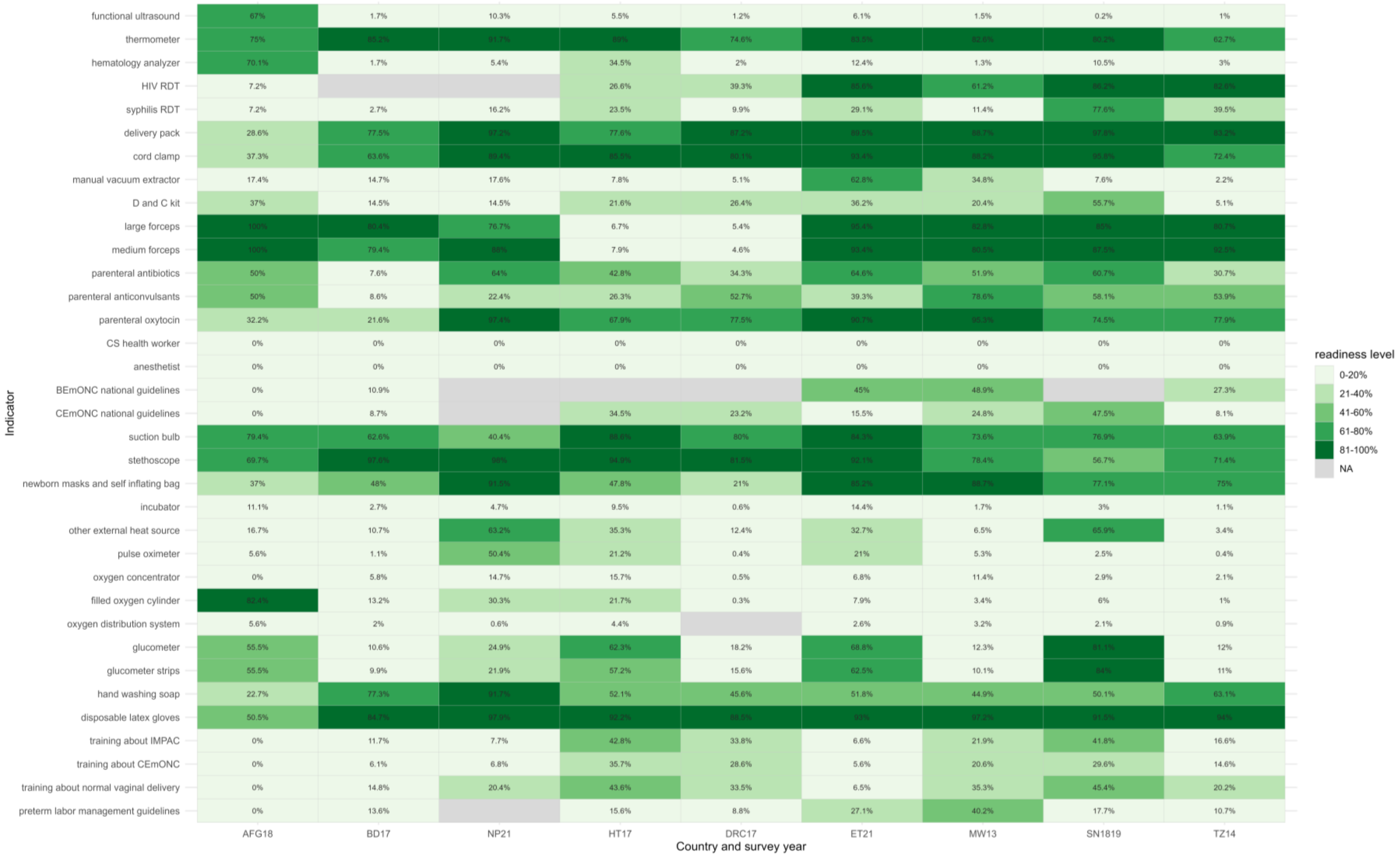

AFG18: Afghanistan 2018-2019; BD17: Bangladesh 2017-2018; NP21: Nepal 2021; HT17: Haiti 2017; DRC17: Democratic Republic of the Congo 2017-2018; ET21: Ethiopia 20121-2022; MW13: Malawi 2013-2014; SN1819: Senegal 2018 and 2019; TZ14: Tanzania 2014-2015;

RDT: rapid diagnostic test; BEmONC: Basic Emergency Obstetrics and Newborn Care; CEmONC: Comprehensive Emergency Obstetrics and Newborn Care; IMPAC: Integrated Management of Pregnancy and Childbirth; NA: not applicable

Figure S5. Heatmap of facility readiness for level 3 facilities by country

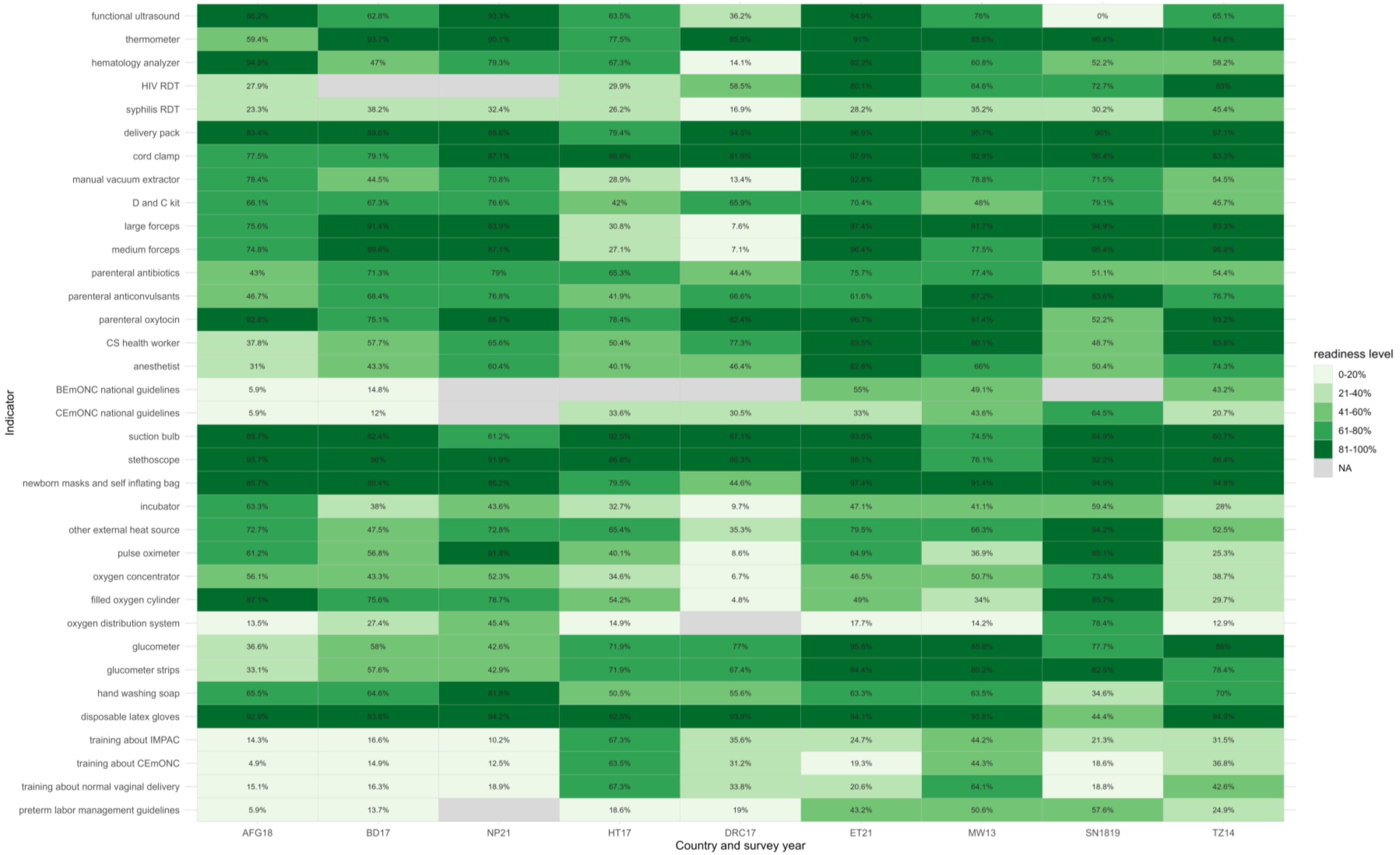

AFG18: Afghanistan 2018-2019; BD17: Bangladesh 2017-2018; NP21: Nepal 2021; HT17: Haiti 2017; DRC17: Democratic Republic of the Congo 2017-2018; ET21: Ethiopia 20121-2022; MW13: Malawi 2013-2014; SN1819: Senegal 2018 and 2019; TZ14: Tanzania 2014-2015;

RDT: rapid diagnostic test; BEmONC: Basic Emergency Obstetrics and Newborn Care; CEmONC: Comprehensive Emergency Obstetrics and Newborn Care; IMPAC: Integrated Management of Pregnancy and Childbirth; NA: not applicable

Figure S6. Differences in overall readiness indexes by antenatal corticosteroids utilization for level 2 and level 3 facilities*

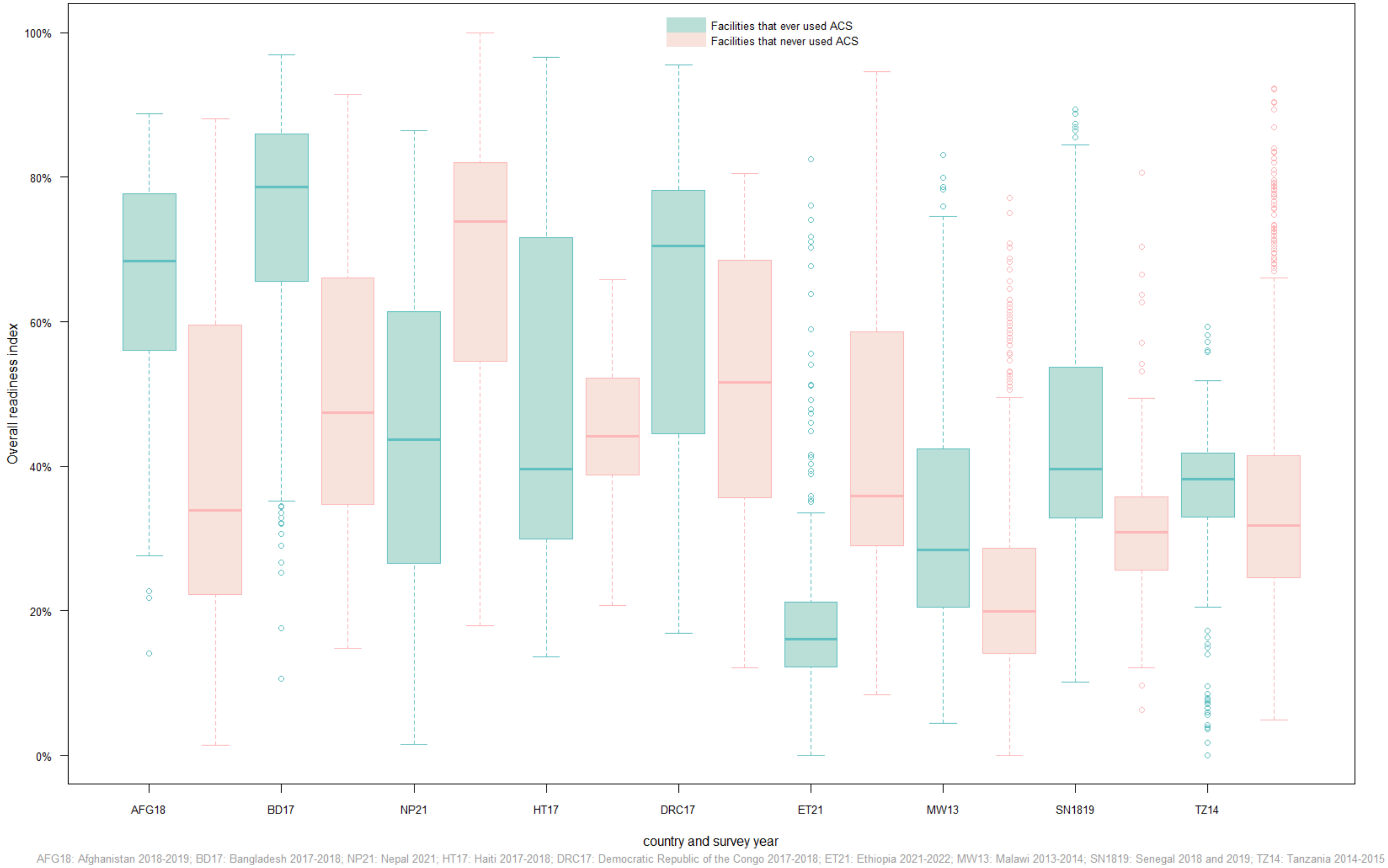

* Level 1 facilities were excluded as none of the level 1 facilities across nine countries had provided ACS.

Figure S7. Differences in overall readiness indexes by antenatal corticosteroids utilization for level 2 facilities

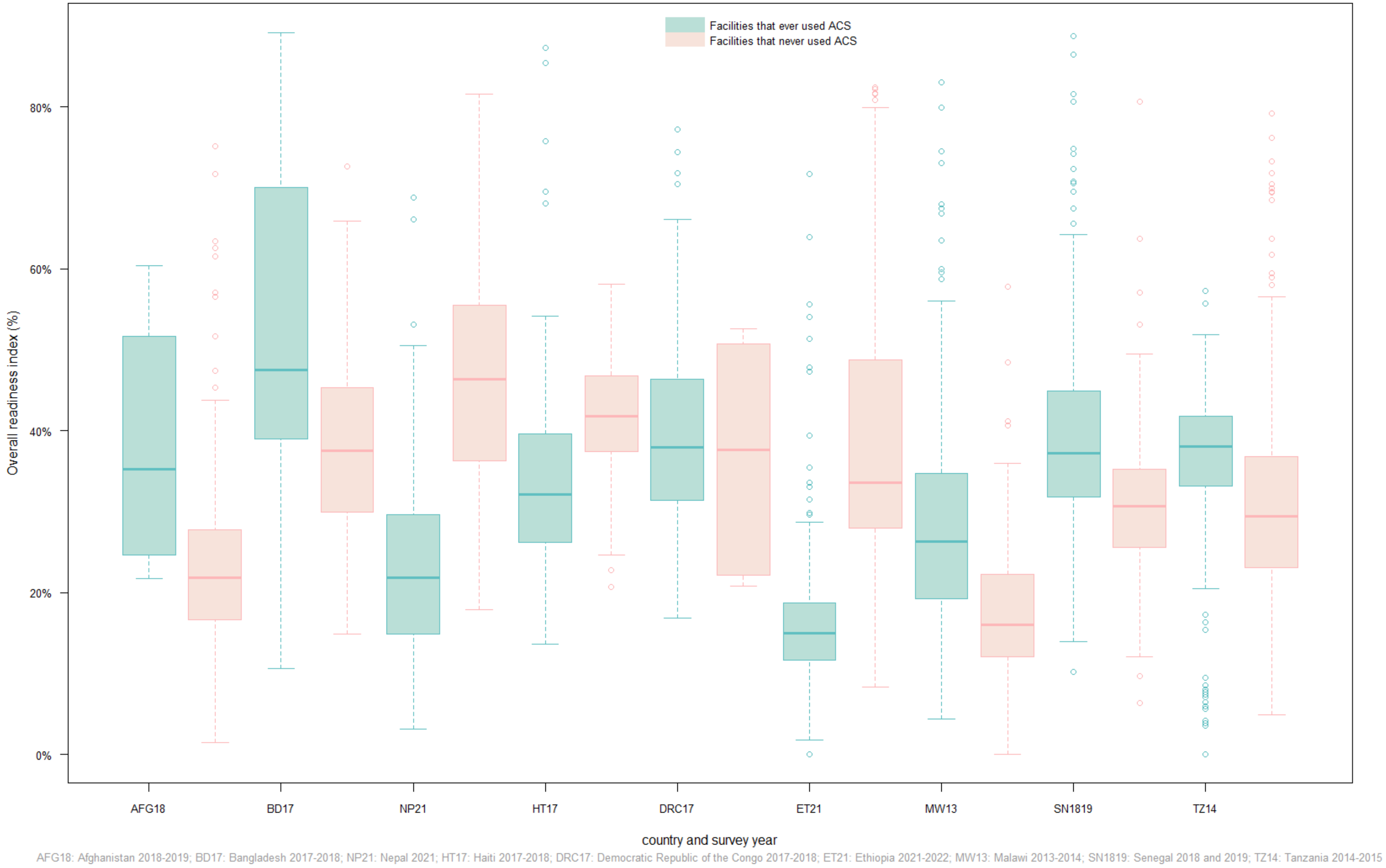

Figure S8. Differences in overall readiness indexes by antenatal corticosteroids utilization for level 3 facilities

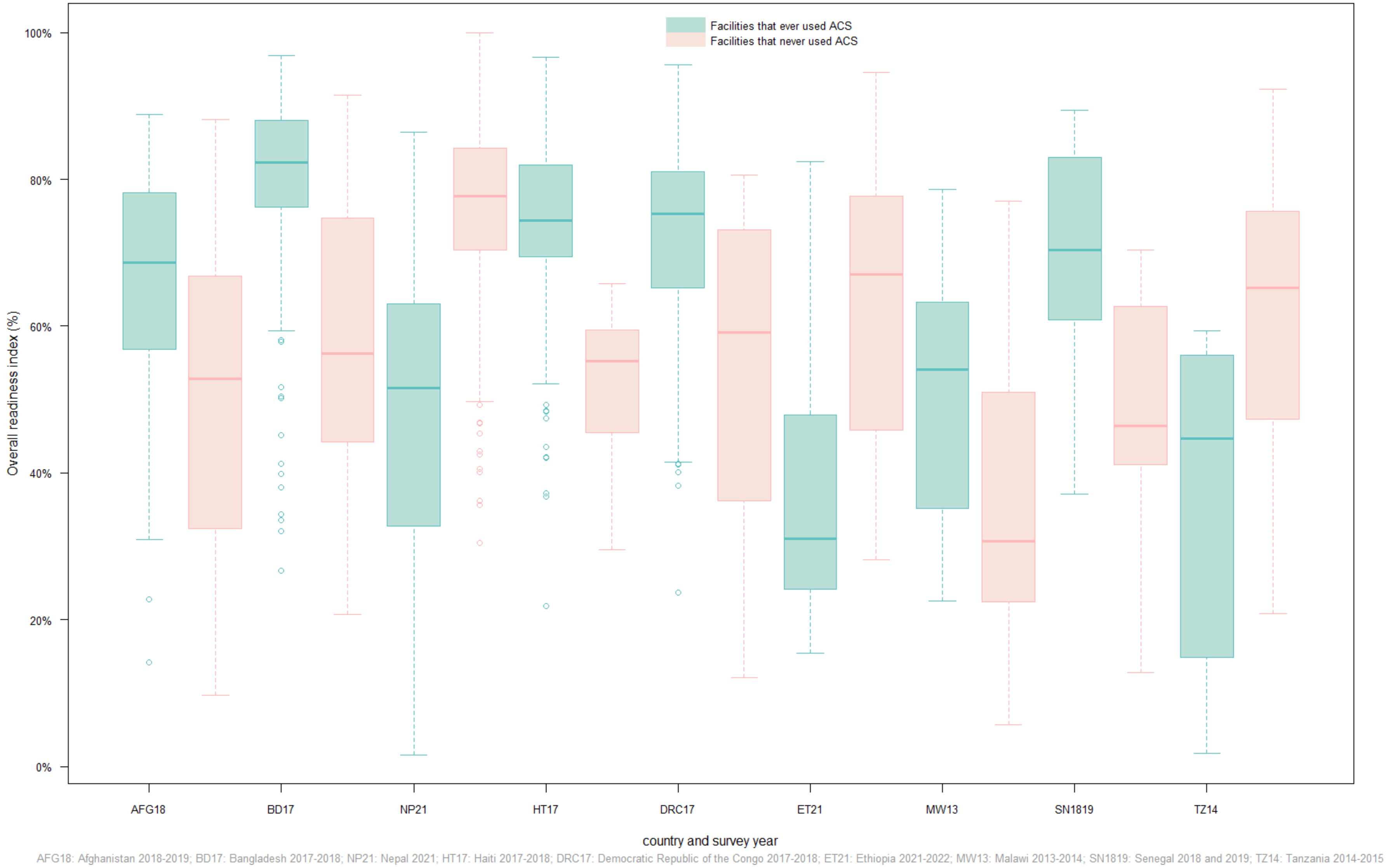

Supplement: Online Supplementary Document [file jogh-15-04149-s001.pdf]
